# What makes a successful species? Traits facilitating survival in altered tropical forests

**DOI:** 10.1186/s12898-017-0135-y

**Published:** 2017-06-28

**Authors:** Mareike Hirschfeld, Mark-Oliver Rödel

**Affiliations:** Department Diversity Dynamics, Museum für Naturkunde Berlin-Leibniz Institute for Evolutionary and Biodiversity Science, Invalidenstraße 43, 10115 Berlin, Germany

**Keywords:** Forest degradation, Frogs, Life-history traits, Adaptation, Extinction risk, Tropics

## Abstract

**Background:**

Ongoing conversion, disturbance and fragmentation of tropical forests stress this ecosystem and cause the decline or disappearance of many species. Particular traits have been identified which indicate an increasing extinction risk of a species, but traits facilitating survival in altered habitats have mostly been neglected. Here we search for traits that make a species tolerant to disturbances, thus independent of pristine forests. We identify the fauna that have an increasing effect on the ecosystem and its functioning in our human-dominated landscapes.

**Methods:**

We use a unique set of published data on the occurrences of 243 frog species in pristine and altered forests throughout the tropics. We established a forest dependency index with four levels, based on these occurrence data and applied Random Forest classification and binomial Generalized Linear Models to test whether species life history traits, ecological traits or range size influence the likelihood of a species to persist in disturbed habitats.

**Results:**

Our results revealed that indirect developing species exhibiting a large range size and wide elevational distribution, being independent of streams, and inhabiting the leaf litter, cope best with modifications of their natural habitats.

**Conclusion:**

The traits identified in our study will likely persist in altered tropical forest systems and are comparable to those generally recognized for a low species extinction risk. Hence our findings will help to predict future frog communities in our human-dominated world.

**Electronic supplementary material:**

The online version of this article (doi:10.1186/s12898-017-0135-y) contains supplementary material, which is available to authorized users.

## Background

The anthropogenic conversion of natural environments, in particular of forest habitats, is a major threat to tropical biodiversity [1]. Beside the intensive loss of forest cover [2], fragmentation of the pristine remnants further affects species [3] and limits their ability to move into adequate areas. Thus the ability to cope with altered landscapes is crucial for the persistence of a species, especially in the face of climate change.

Numerous empirical and comparative approaches on species response to environmental changes and studies relating species properties to their extinction risk were conducted on invertebrates e.g. [4–6] as well as vertebrates e.g. [7–10]. However, the general pattern which leads to the persistence of some species but the decrease or loss of other species due to forest disturbances is not fully understood. In different taxonomic groups some life-history and ecological traits show parallel patterns in their response to forest alteration, e.g. small range size [8, 10, 11] or low fecundity [12, 13] that lead to higher extinction risks. Whereas other traits, like body size exhibit a fuzzy prediction of a species’ risk to decline in fragmented habitats [summary in 14]. The susceptibility of species is not determined by a single trait, but by a combination of properties which lead to a species-specific extinction risk [15–17]. So far, the majority of studies have focused on species affected by environmental changes and filter for traits increasing the extinction risk. Species not responding to habitat alterations and the traits required for their persistence in disturbed landscapes are frequently neglected. However, those species remaining are of high interest as they will make up the majority of the fauna in our human-dominated world and thus have an increasing effect on ecosystems and their functioning [18, 19].

Frogs are strongly influenced by their environment and the degradation and conversion of natural forests is one major cause for their current global decline [20–22]. However, not all species are affected by degradation or fragmentation [23–25] and a set of life-history or ecological traits is assumed to reduce their susceptibility [8, 26].

In this study, we search for factors allowing a species to be independent of pristine areas and thus permitting their occurrence in degraded and disturbed forests, which are the dominant tropical habitats now and in future [27]. We use a unique data set comprising published records on frog species occurrences in tropical forests, forest fragments and more intense altered landscapes such as plantations or settlements. For these species we gathered life-history (e.g. body size, clutch size) and ecological traits (e.g. habitat use) as well as distribution data, which are known to affect the susceptibility of species in general [8, 26, 28] and thus might likewise influence a species response to forest degradation. We ask whether these candidate traits could predict the forest dependency of tropical frog species and whether a particular set of traits makes species less vulnerable to changes in their natural habitat and decreases their risk of extinction.

## Methods

### Data acquisition

We combined a comprehensive data set on anuran occurrences across tropical forests and human altered forest habitats with detailed information on species traits. To cover all research published on anuran distribution in pristine versus altered environments in the tropics, we did a comprehensive literature research using Google, Google Scholar, Web of Science and data bases included therein (January to August 2013). Queries using different combinations of appropriate keywords (e.g. frog, amphibian, anuran, disturbance, alteration, fragmentation, logging etc.) were applied to all data bases. Appropriate data sets covered a description of the study sites and information on the presence (and absence) of each species in the different habitat types. In addition to already published studies we added our own data on anuran occurrences from the forest zone of Cameroon (M. Hirschfeld et al. unpublished data). The survey amounted to 61 studies (see Additional file 1: anuran distribution references) covering all continents that include a tropical climate: Africa, Asia, Central- and South-America, and Australia with a total of more than 750 different anuran taxa. For our analysis we only included records with species level identifications. Species names were checked and updated if necessary according to Frost [30]. If a taxonomic name could not be unambiguously assigned to a valid species, i.e. due to cryptic species complexes, the record was not included. This resulted in a data set with 672 species.

For each valid species from the occurrence data set, its life-history and ecological traits (hereafter referred to as traits) were gathered using published literature, suitable data bases reviewed by specialists, and further web resources (see Additional file 2: anuran traits references). Additionally we included our own unpublished data, collected either in the field or from museum specimens (Museum für Naturkunde Berlin, e.g. body size, ripe eggs in female ovaries). Traits collected and used in the analysis comprised information on species distribution, morphology, biology, and ecology. We also noted the geographic (i.e. continent) and phylogenetic (family) origin of each species (see Table 1 for details). As we only considered species for our analysis where at least information on body size (either male or female) was available, the data set was reduced further to 619 species.

**Table 1:** Life-history and ecological traits used in the study

Trait	Definition	Scale	Unit/level
Range size^a^	Natural area of occurrence	Ratio	km^2^
Elevation	Min. and max. elevation in the entire area of occurrence	Ratio	m asl
SVL male/female	Body length, measured as snout vent length	Ratio	mm
Dimorphism	Calculated as male divided by female body size	Ratio	Proportion
Clutch size	Maximal number of total eggs deposited or maximal number of ripe eggs in the uteri of dissected females	Ratio	#
Clutch size class	Clutch sizes assigned to size classes	Ordinal	Ten size classes, see “Methods” for details
Reproduction	Development	Nominal	Direct, indirect
Adult habitat	Habitat where adults are usually encountered, perch height	Nominal	Aquatic, semi-aquatic, fossorial, litter (<1 m), semi-arboreal (1–3 m), arboreal (>3 m)
Larval habitat	Habitat where the larvae develop	Nominal	None (direct development), terrestrial, semi-aquatic, lentic, lentic and lotic, lotic, phytotelmata (plant associated water bodies, e.g. tree holes, bromeliad tank), skin^b^
Egg deposition	Habitat where the eggs are deposited	Nominal	Terrestrial, semi-terrestrial, aquatic, arboreal, skin^b^
Family	Taxonomic origin, affiliation to family	Nominal	Anuran families according to Frost [30]
Region of origin	Broad geographic region (i.e. continent)	Nominal	

Given is the trait, its definition, the scale of measurement, and the unit (ratio) or levels (nominal, ordinal) of the respective trait

^a^Range size according to the IUCN Red List [29] or, if not available, for West African species to the calculated environmental niche model [70]

^b^Carried in or on adult male or female

### Data preparation

Some of the collected trait data required processing for subsequent analysis. We used the elevational range calculated as the difference from the maximum to the minimum elevation where a species is known to occur. Regarding body size, we used the maximum body length known per species and sex or, if not available, mean values plus standard deviation. Only if maximum and/or standard deviation were not available, mean or single values were used. We supplemented the data set with sexual dimorphism, calculated as male divided by female body size. Clutch size was only available for a subset of species (345). The available data on clutch sizes were grouped objectively into ten size classes (A: 4–98, B: 100–265, C: 290–549, D: 563–905, E: 979–1652, F: 1900–3320, G: 3607–6701, H: 8357–12940, I: 17000–25000, J: 36100–40000) and species without information on clutch size were subsequently assigned to a class based on body size (see Additional file 3: clutch size classes for more details).

Studies included in our analyses focused on the comparison of anuran distribution among various landscapes. Hence, broader habitat categories were necessary to combine the results within one analysis. Based on all information available we chose three major habitat categories along a human altered degradation gradient: forest, secondary growth, and non-forest. The habitat category “forest” comprises primary forests, primary forest fragments, and selectively logged or exploited areas; “secondary growth” subsumes secondary forests, edges of primary forests, abandoned plantations (>5 years) and agricultural habitats with remaining forests (e.g. shaded coffee plantations); non-forests comprise simple structured plantations (single strata), pasture or inhabited areas such as villages. Categorization was realized in accordance with comparative studies [4, 31, 32]. However, in consideration of the modified forest types examined in our data set, slight adaptations and a reduction of categories were necessary. We only took species into account which had information on the presence and absences in these major habitat categories. If a species was detected in several studies, its single occurrence per habitat category (although absent in other studies) was crucial to assign the species to that habitat type. Combining this reduced data set with the available trait data, the final data set amounted to 243 different species with only a few gaps for some traits. As multivariate statistics often require complete data sets, missing values in the trait data set were replaced by dummy variables. This prevents a high loss of information by excluding a trait or a species. For traits with a ratio scale we used the mean, and for traits with a nominal scale the level which occurred most often (compare Table 1). Numbers of required dummy variables in the final data set: range size = 2 (mean = 1,795,153 km^2^), elevational range: 35 (1217.6 m), snout-vent length (SVL) males: 4 (10.5 mm), SVL females: 21 (18 mm), reproductive mode: 1 (most frequent: indirect development), adult habitat: 2 (litter); larval habitat: 11 (lentic), egg deposition site: 34 (aquatic). All analyses were conducted with the completed data set (see Additional file 4).

**Fig. 1:**
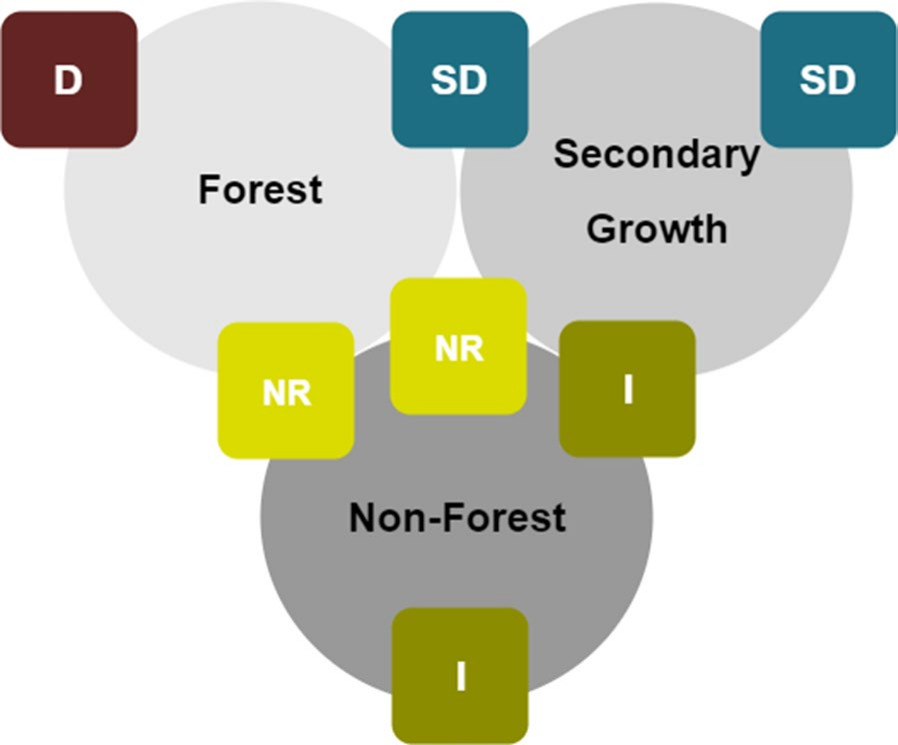
Forest dependency index. A forest dependency index (FDI) was established based on species occurrences in three major habitat categories (forest, secondary growth, non-forest); FDI: *D* dependent, solely detected in forests, *SD* slightly dependent, species occurring in forests and habitats with secondary growth, *I* forest independent species, occuring not in primary forests, i.e. only in habitats with secondary growth and/or non-forested habitats, *NR* species with no response, occurring in all three habitat categories or forest and non-forest habitats

Based on the species occurrences in the three major habitat categories, a forest dependency index (FDI) with four levels was established (Fig. 1): dependent species solely detected in forests (D), slightly dependent species occurring in forests and habitats with secondary growth (SD), forest independent species occurring not in primary forests, i.e. only in habitats with secondary growth and/or non-forested habitats (I), and species with no response occurring in all three habitat categories or forest and non-forest habitats (NR).

### Statistical analysis

The distribution and trait data (ratio scale) were non-normal distributed (Shapiro–Wilk test, R package ‘stats’). We thus applied the non-parametric Kruskal–Wallis test and subsequent pairwise Wilcoxon tests with false discovery rate (*fdr*) correction for parameter comparison among species with different forest dependency indices (R package ‘stats’). To filter for species traits explaining the presence or absence of a species in differently degraded habitats and thus their assignment to a particular FDI we performed a Random Forest (RF) classification [33] where 1000 classification trees on bootstrap samples of the data were grown (*randomForest*, R package ‘randomForest’ [34]). The number of candidate variables at each node (*mtry*) was the square root of the total number of variables in the analysis (default setting). To correct for different sample sizes in the training data set, *sampsize* was adjusted according to the minimum sample size per analysis. RF was performed for the whole data set and four subsets, three comparing forest dependent species (D) with one of the other FDIs and a comparison of the groups NR and I. We incorporated all available information for a species in RF, including species distribution (range size, elevation range, region of origin) and seven traits (see Table 1). As families were evenly distributed among the different FDIs (see Additional file 5), the affiliation to a family was excluded from the analysis. Binomial Generalized Linear Models (GLM) were performed to filter for potential traits explaining the habitat dependency of a species (*glm*, R package ‘stats’). Therefore, species not responding to habitat changes (NR) were defined as ‘0’ and compared to forest dependent species (D) as well as forest independent species (I), both defined as ‘1’. Numerical variables (body size and sexual dimorphism) were scaled from 0 to 1. To avoid multi-co-linearity among explaining variables within one model, generalized variance inflation factors (GVIF) were calculated (*vif*, R package ‘car’). Each model contained the covariates: SVL females, sexual dimorphism, clutch size class, larval habitat, adult habitat, reproductive mode, and egg deposition site. After reducing the co-linearity among the explaining variables and eliminating those with a GVIF higher than five [35], the full model only contained: SVL females, sexual dimorphism, clutch size class, and larval habitat. To test for any influence on the forest dependency of species distribution we fitted Generalized Linear Mixed Effect Models (GLMM) with range size and elevational range (both scaled from 0 to 1) as fixed and the region of origin as random factor (*glme*, R package ‘lme4’). Here, a reduction of covariates due to co-linearity was not necessary. Based on the models, we predicted whether a species is either dependent on forest (non-forest) or occurs in all available habitats (≥0.5 for forest, D or non-forest, I; <0.5 for habitat independent species, NR). All statistical analysis were applied using R 3.2.1 [36].

## Results

### Taxonomy

The 243 anuran species included in the analysis belonged to 26 different families. The most common families were Rhacophoridae and Hylidae, the latter representing 10–30% of the species in all forest dependency indices (FDIs). The families were equally distributed among the different FIDs (see Additional file 5), ruling out any phylogenetic influence in the data.

### Species distribution

**Fig. 2:**
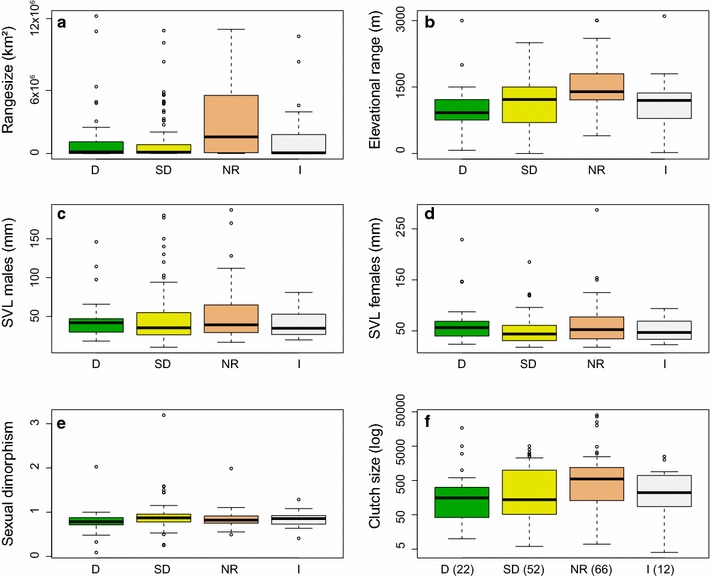
Comparison of distribution patterns and life history traits among anuran species with different forest dependency indices. Given is the range size (**a**), distribution range along the elevation (**b**), the maximum body length of males (**c**) and females (**d**), size dimorphism between sexes (males/females, **e**) and the maximum clutch size (**f**); in addition to the available data, dummy variables were calculated (see “Methods”) and added (**a**–**e**); forest dependency index: *D* dependent (n = 33), *SD* slightly dependent (n = 108), *NR* non-responding (n = 83), *I* forest independent (n = 19), for clutch size (**f**) only measured values are shown, differing sample sizes are given in the graph; see Table 2 for statistical comparisons

**Fig. 3:**
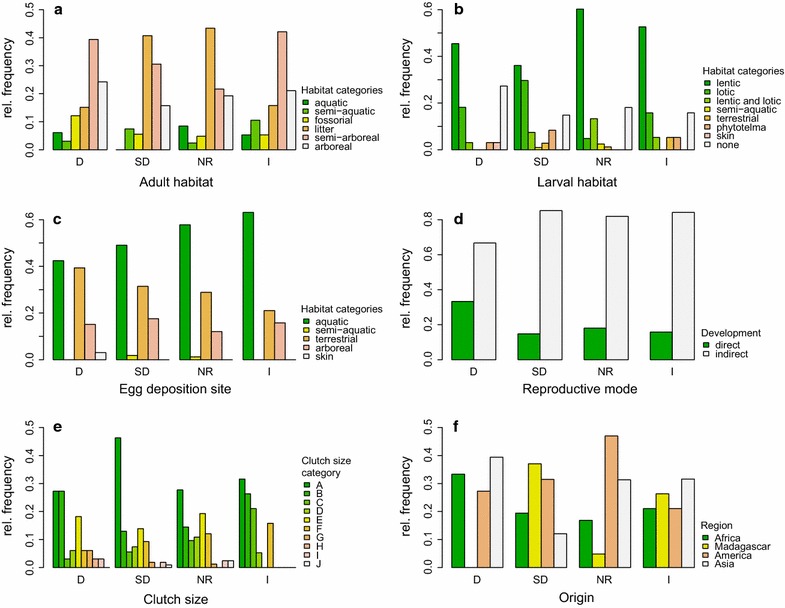
Comparison of life history traits and species’ origin among anuran species with different forest dependency indices. Shown is the relative frequency of a category for adult (**a**) and larval (**b**) habitats, the egg deposition site (**c**), the reproductive mode (**d**), the clutch size (**e**), and the region of origin (**f**); in addition to the available data, dummy variables were calculated (see “Methods”) and added to the data set (**a**–**e**); forest dependency index: *D* dependent (n = 33), *SD* slightly dependent (n = 108), *NR* non-responding (n = 83), *I* forest independent (n = 19); see legends for color codes for each plot separately, y-axis are scaled differently; for details on habitat types and definition see “Methods” and Table 1; see “Results” for statistical comparisons of frequencies

Range sizes ranged from 6.17 to 12,217,676 km^2^ and varied highly within each FDI (Table 1 for information on gathered traits; see Table 2; Fig. 2 for results). It differed significantly between forest dependent species (D) and species not responding to habitat alteration (NR) as well as between species slightly depending on forests (SD) and NR. All FDIs covered species with limited and wide altitudinal distribution (see Fig. 2). NR species had the broadest distribution and differed significantly from the others (see Table 2). Species in the final data set originated from Africa, Madagascar, America and Asia. The indices NR, I and SD comprised species from all four regions, only D was lacking Malagasy species (Fig. 3). The region of origin did not differ significantly between the FDIs (Pearson’s χ^2^ test: χ^2^ = 5.89, df = 3, p = 0.12).

**Table 2:** Distribution pattern and life history traits

Trait	General n = 243		D n = 33		SD n = 108		NR n = 83		I n = 19	
	Mean ± SD	Range	Mean ± SD	Range	Mean ± SD	Range	Mean ± SD	Range	Mean ± SD	Range
a										
Range size (km^2^)^a^	1,795,153 ± 2,784,023	6.17–12,217,676	1,511,311 ± 2,996,433	14.67–12,217,676	1,086,942 ± 2,069,589	6.17–10,932,823	2,850,720 ± 3,163,102	21.62–11,045,631	1,702,603 ± 2,983,042	305.09–10,419,167
Elevational range (m)^a^	1217.60 ± 591.78	1–3100	994.04 ± 582.89	72–3000	1123.0 ± 557.65	1–2500	1446.33 ± 561.84	400–3002	1144.46 ± 652.31	20–3100
SVL males (mm)^a^	47.13 ± 30.25	10.5–187	45.83 ± 26.96	18.4–146	45.90 ± 31.85	10.5–180	50.34 ± 31.29	17.0–187	42.44 ± 21.00	20.0–81
SVL females (mm)^a^	56.37 ± 34.04	18–287	63.06 ± 40.50	24–228.9	50.80 ± 26.95	18–185.0	61.99 ± 40.10	18–287.0	51.90 ± 24.22	23–94.0
Sexual dimorphism^a^	0.86 ± 0.26	0.09–3.19	0.79 ± 0.29	0.09–2.03	0.90 ± 0.31	0.25–3.19	0.83 ± 0.18	0.49–1.99	0.84 ± 0.19	0.41–1.29
Clutch size^b^	1609.62 ± 5186.19	4–40,000	1296.36 ± 3701.47	10–17,000	748.99 ± 1158.77	6–5018	2578.56 ± 7423.93	7–40,000	584.08 ± 823.73	4–2500

Trait	Kruskal–Wallis test			Pairwise Wilcox test (p)						
	χ^2^	df	p	D vs SD	D vs. NR	D vs I	SD vs. NR	SD vs. I	NR vs. I	
b										
Range size (km^2^)^a^	19.72	3	<0.001	0.29	<0.01	0.32	<0.01	0.50	0.07	
Elevational range (m)^a^	21.7	3	<0.0001	0.26	<0.001	0.46	<0.001	0.82	<0.05	
SVL males (mm)^a^	2.67	3	0.45	–	–	–	–	–	–	
SVL females (mm)^a^	5.72	3	0.13	–	–	–	–	–	–	
Sexual dimorphism^a^	8.92	3	<0.05	0.05	0.38	0.38	0.11	0.46	0.71	
Clutch size^b^	6.92	3	0.07	–	–	–	–	–	–	

Given are the respective mean, standard deviation (sd), and range in general, and for each dependency index separately (a) and comparisons of traits between species of different forest dependency indices using the Kruskal–Wallis test and a pairwise Wilcox test with fdr correction as posthoc (b); forest dependency index: D = dependent (n = 33), SD = slightly dependent (n = 108), NR = non-responding (n = 83), I = forest independent (n = 19)

^a^Incorporate calculated dummy variables (see “Methods”)

^b^Only measured values and therewith differing sample sizes: general = 152, D = 22, SD = 52, NR = 66, I = 12; compare Figs. 2 and 3

### Habitat

Overall, most species preferred litter as well as shrubs and lower tree strata (1–3 m) as adult habitat (Fig. 3). Almost 75% of the species belonging to D and I live in trees (categories semi-arboreal and arboreal); SD and NR species were mostly found on the ground. Aquatic habitats were not inhabited by SD species, while the other FDIs covered all types. The habitat use differed slightly among the FDIs (Pearson’s χ^2^ test: χ^2^ = 27.28, df = 15, p = 0.03). Lentic waters constitute 35–60% of the tadpoles’ habitat per FDI (Fig. 3). Lotic waters were of high importance in SD species, but less in other FDIs. All other categories were only sparsely presented, apart from no larval habitat, representing direct developing species. The larval habitat differed significantly between species assigned to different FDIs (χ^2^ = 45.23, df = 21, p = 0.002).

### Body size

Maximum body sizes ranged from 10 to 187 mm for males and from 18 to 287 mm for females, respectively, with a high variation for both sexes within each FDI (see Table 2; Fig. 2). It did not differ between the FDIs (see Table 2). Sexual dimorphism also did not show large differences between the indices, but the comparison between D and SD species showed a trend towards D hosting species with greater dimorphism. As female and male body size were highly correlated (Spearman Rank Correlation: ρ = 0.88, p < 0.0001, n = 243), we only used female body size and dimorphism in subsequent analysis.

### Reproduction

Clutch size varied between 4 and 40,000 eggs and did not differ between the FDIs (see Table 2; Fig. 2). Independent of the FDI, most clutches were in the first two size classes (4–98 and 100–265 eggs). Species belonging to I did not have clutches greater than 6700 eggs. The clutch size measured in categories likewise did not differ significantly between the FDIs (Pearson’s χ^2^ test: χ^2^ = 34.96, df = 27, p = 0.14). Most species deposited their eggs in aquatic habitats (see Fig. 3). The second most common habitat was terrestrial, followed by arboreal deposition sites. There were no significant differences in egg deposition site between the FDIs (χ^2^ = 11.52, df = 12, p = 0.48). Almost 80% of the investigated species showed a biphasic development with free swimming tadpoles (see Fig. 3); D species had the highest proportion of direct developers (>30%). The reproductive mode did not differ significantly between the FIDs (χ^2^ = 5.89, df = 3, p = 0.12).

Classification by RF on the whole data set resulted in an overall error rate of 50.2%, the misclassification per FDI varied between 42.2 and 94.4% (see Table 3). Classification of subsets performed better, with an overall error rate of 20.7% (D vs. NR), 22.0% (D vs. I), 30.4% (NR vs. I), and 40.4% (D vs. I). Range size was important in all, sexual dimorphism and elevational range in four, and clutch size category in three models (Table 4).

**Table 3:** Confusion matrices of Random Forest analysis

	D	I	NR	SD	CE (%)	OE (%)
Complete data set						50.2
D	15	4	9	5	54.5	
I	5	1	7	6	94.4	
NR	12	6	48	17	42.2	
SD	9	12	29	57	47.2	
Subset D vs. SD						22.0
D	17	–	–	16	48.5	
SD	15	–	–	93	12.0	
Subset D vs. NR						20.7
D	21	–	12	–	36.4	
NR	12	–	71	–	14.5	
Subset D vs. I						40.4
D	23	10	–	–	30.3	
I	11	8	–	–	57.9	
Subset I vs. NR						30.4
I	–	9	10	–	52.6	
NR	–	21	62	–	25.3	

Confusion matrices with per class error (CE) rate and overall error (OE) rate per Random Forest analysis (complete data set and different subsets); analysis were performed with *ntree* = 1000, *mtry* = 3 and *sampsize* adjusted to the smallest sample size (R package ‘randomForest’); forest dependency index: *D* dependent (n = 33), *SD* slightly dependent (n = 108), *NR* non-responding (n = 83), *I* forest independent (n = 19)

**Table 4:** Importance of each variable in Random Forest analysis

Variable	Complete	D vs. SD	D vs. NR	D vs. I	I vs. NR
SVL females	*6.73*	3.41	*3.42*	2.15	2.08
Adult habitat	4.70	3.23	*3.64*	0.91	2.37
Sexual dimorphism	*9.02*	*4.00*	2.98	*2.63*	*2.59*
Clutch size category	6.15	*3.49*	3.39	*4.20*	*2.63*
Egg deposition site	1.13	1.04	0.46	0.50	0.48
Reproductive mode	0.14	0.47	0.07	0.10	0.14
Larval habitat	3.46	2.63	2.10	0.89	1.12
Region	4.54	*4.95*	2.62	2.11	1.58
Range size	*12.52*	*5.91*	*7.52*	*3.27*	*3.06*
Elevational range	*8.59*	3.37	*6.81*	*2.23*	*2.87*

Importance of each variable per Random Forest analysis (complete data set and different subsets); the four most important variables contributing to the classification are in italics; analysis were performed with *ntree* = 1000, *mtry* = 3 and *sampsize* adjusted based on the smallest sample size for each analysis respectively (R package ‘randomForest’); forest dependency index: *D* dependent (n = 33), *SD* slightly dependent (n = 108), *NR* non-responding (n = 83), *I* forest independent (n = 19)

Generalized linear models (Table 5) revealed larval habitat and clutch size class as important factors explaining the dependency to forests (D vs. NR species) with the development in lotic waters being significant and clutches of class G (3607–6701 eggs) being almost significant. Based on this model, 77% of all species could be correctly assigned to the original FDIs (matches: D: 12, n = 33; NR: 77, n = 83; sample size from original data). The model for forest independent species (I vs. NR species) revealed likewise larval habitat as being important with development in lotic waters being significant. The model assigned 82% of all the species correctly to the FDI derived from field observation (I: 4, n = 19; NR: 81, n = 83). Generalized Linear Mixed Models (Table 5) fitted with species distribution revealed elevational range as being important factors when comparing both, D and NR as well as I and NR species (the latter barely non-significant). The model contrasting D and NR species assigned 78% of the species to correct FDIs (D = 13; NR = 77), based on the model comparing I and NR species 71% were correctly classified compared to the original FDIs (I = 0; NR = 83). Results of the different approaches confirm each other at least partly: RF vs. GLM: forest dependent species (D): classification overlap of 72%, matches: D: 9; NR: 74; forest independent species (I): 75%, I = 5; NR: 71; RF vs. GLMM: D: 84%, D = 16, NR = 81; I: 71%, I = 0, NR = 72; GLM vs. GLMM: D: 77%, D = 5, NR = 84; I: 94%, I = 0; NR = 96.

**Table 5:** Effects of species traits and distribution on habitat dependency

	Forest dependent species (D vs. NR)				Non-forest species (I vs. NR)			
	Estimate	Std. error	z	p	Estimate	Std. error	z	p
GLM on species traits								
Intercept	0.085	1.47	0.058	0.95	−2.98	1.37	−2.18	*0.03*
SVL females	−0.08	2.70	−0.03	0.98	0.05	4.59	0.01	0.99
Sexual dimorphism	−3.73	3.11	−1.20	0.23	2.71	2.83	0.96	0.34
Larval habitat								
Lentic/lotic	−17.13	1852.28	−0.009	0.99	−1.13	1.30	−0.87	0.38
Lotic	1.66	0.77	2.16	*0.03*	3.00	1.34	2.23	*0.03*
None	0.39	0.64	0.61	0.54	0.26	0.91	0.28	0.78
Phytotelma	18.95	6522.64	0.003	0.99	22.54	10,750	0.002	0.99
Semi-terrestrial	−17.22	4611.48	−0.004	0.99	−16.31	6635	−0.002	0.99
Skin	19.79	6522.64	0.003	0.99	–	–	–	–
Terrestrial	−0.08	2.70	−0.03	0.98	2.50	1.67	1.49	0.14
Clutch size class								
B	0.67	0.67	1.01	0.31	1.14	0.86	1.34	0.18
C	−0.57	1.22	−0.47	0.64	1.73	1.07	1.61	0.11
D	−0.19	1.02	−0.19	0.85	0.20	1.36	0.15	0.88
E	−0.02	0.79	−0.03	0.98	−18.48	2343	−0.01	0.99
F	−0.10	1.10	−0.09	0.93	0.85	1.32	0.64	0.52
G	3.16	1.86	1.70	0.09	−17.69	10,750	−0.002	0.99
H	37.40	6780.54	0.01	0.99				
I	0.59	1.57	0.38	0.71	−17.37	7585	−0.002	0.99
J	−16.74	3995.08	−0.004	0.99	−16.46	7482	−0.002	0.99
GLMM on species distribution								
Fixed effects								
Intercept	1.23	0.68	1.80	0.07	−0.004	0.69	−0.006	0.99
Range size	−1.59	1.13	−1.41	0.16	−1.18	1.38	−0.86	0.39
Elevational range	−4.97	1.45	−3.44	*0.0006*	−3.03	1.56	−1.85	0.06

Binomial models for forest dependent and non-forest species were conducted and full models (*glm*, R package ‘stats’; *glme*, R packages ‘lme4’) after eliminating multicollinearity (*vif*, R package ‘car’) are presented; Generalized Linear Model (GLM): variables included: SVL females, sexual dimorphism, clutch size class, larval habitat; removed due to co-linearity: adult habitat, reproductive mode, and egg deposition site; Generalized Linear Mixed Model (GLMM): range size and elevation range as fixed and region of continent random factors (no co-linearity among explaining variables); significant effects are in italics; *D* forest dependent species (n = 33), *NR* non-responding species (n = 83), *I* forest independent species (n = 19)

## Discussion

Geographic range size has been identified as a vital factor predicting a species’ susceptibility and extinction risk, including birds [11], mammals [10, 16], and amphibians [8, 37]. Species tolerating a wide range of abiotic factors, different habitats [38], or not responding to forest degradation (this study) likewise have the widest distribution. Here, we assign species to one of four levels of forest dependency, according to their occurrence in habitats with differently strong disturbance. Species belonging to D (forest dependent) depend on pristine forests, species assigned to the other categories (NR, I, or SD) can cope with habitat disturbances to different extents. We determined the most important traits explaining the forest dependency of a species using RF classification, GLM, and GLMM techniques. Since range size and extinction risk or habitat breadth might directly depend on each other, making it a single criterion to assess species as critically endangered in the IUCN Red List [29], we excluded it in the GLM filtering for species traits, but analyzed it separately (GLMM). Here, however, only elevational range was important for distinguishing NR from D and NR from I species. This is consistent with previous results where a wide altitudinal distribution decreases a species’ vulnerability [9, 39, 40], as such species are naturally adapted to varying environmental factors (e.g. vegetation, climate) and hence might also cope better with changes of these factors caused by forest disturbances.

Body size is a central trait, usually correlated with factors such as population size, range size, clutch size or rate of exploitation, all influencing the extinction risk of a species [41–43]. It was thus typically taken into consideration when estimating a species’ susceptibility. With increasing body size, studies revealed an increase (amphibians: [39], mammals: [41], birds: [44]), or, as in our data, no change in the extinction risk (amphibians: [28], birds: [40], bats: [45]). These converse results emphasize the complex effects of body size and explain the variation in its influence on the vulnerability of species, differing with study systems [14] but also with the source of extinction risk [46]. According to our results, neither body size nor sexual size dimorphism seem to influence forest dependency.

Although the number of offspring explains the extinction risk in several taxa [12, 13], traits related to reproduction only had minor effects on degradation susceptibility of a frog species in our data set. Species belonging to I, however, do not deposit bigger clutches (separating I from NR species in RF). This could either be related to the larger number of I species using flowing, not stagnant, waters as larval habitat and the fact that stream breeders tend to have bigger eggs and thus smaller clutches [47], or to the absence of bigger females, depositing larger clutches (see Figure in Additional file 3) in I. A higher percentage of direct developers among forests dependent species (this study, but see [48, 49]) and an increased extinction risk of ovoviviparous anuran species in general ([8], but see [50]) can be explained by the required moist microhabitat for a direct development [51], available in pristine forests, but not necessarily in degraded or fragmented habitats [52, 53].

A species’ microhabitat preferences affect its vulnerability, i.e. the availability of breeding sites, particular soil conditions or vegetation structure can be crucial for the presence of an amphibian species [e.g. 49, 54, 55]. Modified forests are accompanied by an open canopy which facilitates the growth of herbaceous strata and leads to an advantageous humid microclimate for some leaf-litter anurans. This structured understory, including downed woody debris, has been identified as an important habitat feature for amphibian populations in altered forests [56, 57] and explains the increase of ground dwelling species among degradation tolerant species [25, this study]. Degradation with an accompanying loss of canopy cover generates the most prominent microclimatic shifts in the mid-story, forming the upper strata after disturbances. The resulting decreased humidity, stronger temperature extremes, and increased solar radiation [58–60] have adverse effects on amphibians and explain the high number of semi-arboreal species in our study being forest dependent and the low number being degradation tolerant.

Forest degradation negatively impacts riparian habitats for amphibians by decreasing the amount of woody debris or leaf litter, resulting in less dissolved organic carbon [61] and by a reduction of the canopy cover, leading to higher temperatures and solar radiation [62, 63]. These unfavorable changes explain the higher number of stream breeders among species prone to degradation (this study) and the higher susceptibility of species dependent on lotic breeding sites [54] and riparian species in general [39, 50]. Although forest degradation potentially cause similar changes in lentic habitats, pond breeding amphibians might be less vulnerable or, due to different life-history strategies, even benefit from the consequences: higher temperatures for example increase the developmental rate [64, 65] and higher solar radiation favors the growth of algae [62], the primary food resource for many pond dwelling tadpoles. Compared to species not responding to habitat changes, also a higher number of non-forest species strongly depend on rivers for their tadpole development. These species might be already accustomed to open riparian habitats and thus do not suffer from the prevailing conditions like species occurring in all habitat types.

When contrasting the classification of RF, GLM, and GLMM based on the comparisons D vs. NR, ten species were always wrongly assigned. For example two species, known to occur in strongly degraded habitats [66] and to reproduce in artificial ponds [67] were assigned to D but predicted to belong to NR. Hence the models predicted the species correctly and only the incorporated information from the field was limited and did not cover the occurrences in altered habitats.

## Conclusions

Generalist species were identified as the winners in human-dominated landscapes [18, 68], but particular traits facilitating this adaptation were not yet determined. Our pan-tropical approach revealed that the dependency to forested habitats is explained by traits similar to those generally recognized for high species extinction risk. Indirect developing species exhibiting a big range size, wide elevational range, being independent of streams, and inhabiting the leaf litter are less prone to modifications of their natural habitats. As the effect of a particular trait on the vulnerability of a species might differ among threats [17, 69] and study scales (local vs. global), the generality of our results needs to be treated with caution. However, our findings point to the traits persisting in degraded habitats and thus help to identify future frog communities in our human-dominated world.

## Additional files

**Additional file 1.** Anuran distribution references. References to studies appropriate for the study. Respective data were incorporated in the primary data set on anuran occurrence in different habitat types.

**Additional file 2.** Anuran traits references. References to journal articles, books and web resources containing information on species traits for the species included in the primary data set on anuran occurrence.

**Additional file 3.** Clutch size classes. Additional methods describing the objective grouping of clutch sizes.

**Additional file 4.** Final data set. Given is the species, the traits looked at and the forest dependency index (FDI) derived from the occurrence data. Dummy variables are highlighted in grey. See Table 1 for information on the respective traits.

**Additional file 5.** Affiliation to different anuran families. Number and relative frequency of species belonging to a particular family per forest dependency index.
