# Supplementary material for: What makes a successful species? Traits facilitating survival in altered tropical forests
Source: BMC Ecol. 2017 Jun 28;17:25. doi: 10.1186/s12898-017-0135-y (PMC5490239; doi:10.1186/s12898-017-0135-y)
Supplement: Supplementary file 1 — Additional file 1. Anuran distribution references. References to studies appropriate for the study. Respective data were incorporated in the primary data set on anuran occurrence in different habitat types. [file 12898_2017_135_MOESM1_ESM.docx]

**Additional file 1. Anuran distribution references**

References to studies appropriate for the study. Respective data were incorporated in the primary data set on anuran occurrence in different habitat types.

Adum, G. B., M. P. Eichhorn, W. Oduro, C. Ofori-Boateng & M.-O. Rödel (2013): Two-stage recovery of amphibian assemblages following selective logging of tropical forests. Conservation Biology 27: 354-363.

Akani, G. C., E. Politano & L. Luiselli (2004): Amphibians recorded in forest swamp areas of the River Niger Delta (southeastern Nigeria), and the effects of habitat alteration from oil industry development on species richness and diversity. Applied Herpetology 2: 1-22.

Bell, K. E. & M. A. Donnelly (2006): Influence of forest fragmentation on community structure of frogs and lizards in Northeastern Costa Rica. Conservation Biology 20: 1750-1760.

Bickford, D., T. How Ng, L. Qie, E. P. Kudavidanage & C. J. A. Bradshaw (2010): Forest fragment and breeding habitat characteristics explain frog diversity and abundance in Singapore. Biotropica 42:119-125.

Cabrera-Guzmán, E. & V. H. Reynoso (2012): Amphibian and reptile communities of rainforest fragments: minimum patch size to support high richness and abundance. Biodiversity and Conservation 21: 3243-3265.

Cáceres-Andrade, S. P. & J. N. Urbina-Cardona (2009): Esamblajes de anuros de sistemas productivos y bosques en el piedemonte Llanero, Departamento del Meta, Colombia. Caldasia 31: 175-194.

Campos, V. A., F. H. Oda, L. Juen, A. Barth & A. Dartora (2013): Composition and species richness of anuran amphibians in three different habitat in an agrosystem in Central Brazilian Cerrado. Biota Neotropica 13: 124-132.

Cortés, A. M., M. P. Ramírez-Pinilla, H. A. Suárez & E. Tovar (2008): Edge effects on richness, abundance and diversity of frogs in Andean cloud forest fragments. South American Journal of Herpetology 3: 213-222.

da Silva, F. R. & d. C. Rossa-Feres (2011): Influence of terrestrial habitat isolation on the diversity and temporal distribution of anurans in an agricultural landscape. Journal of Tropical Ecology 27: 327-331.

de Lima, M. G. & C. Gascon (1999): The conservation value of linear forest remnants in central Amazonia. Biological Conservation 91: 241-247.

Dixo, M. & M. Martins (2008): Are leaf-litter frogs and lizards affected by edge effects due to forest fragmentation in Brazilian Atlantic forest? Journal of Tropical Ecology 24: 551-554.

Ernst, R., K. E. Linsenmair & M.-O. Rödel (2006): Diversity erosion beyond the species level: dramatic loss of functional diversity after selective logging in two tropical amphibian communities. Biological Conservation 133: 143-155.

Ernst, R. & M.-O. Rödel (2005): Anthropogenically induced changes of predictability in tropical anuran assemblages. Ecology 86: 3111-3118.

Ernst, R. & M.-O. Rödel (2008): Patterns of community composition in two tropical tree frog assemblages: separating spatial structure and environmental effects in disturbed and undisturbed forests. Journal of Tropical Ecology 24: 111-120.

Faria, D., M. L. Barradas Paciencia, M. Dixo, R. R. Laps & J. Baumgarten (2007): Ferns, frogs, lizards, birds and bats in forest fragments and shade cacao plantations in two contrasting landscapes in the Atlantic forest, Brazil. Biodiversity and Conservation 16: 2335-2357.

Ficetola , G. F., D. Furlani, G. Colombo & F. de Bernardi (2008): Assessing the value of secondary forest for amphibians: *Eleutherodactylus* frogs in a gradient of forest alteration. Biodiversity and Conservation 17: 2185-2195.

Fredericksen, N. J. & T. S. Fredericksen (2002): Terrestrial wildlife responses to logging and fire in a Bolivian tropical humid forest. Biodiversity and Conservation 11: 27-38.

Furlani, D., G. F. Ficetola, G. Colombo, M. Ugurlucan & F. de Bernardi. (2009): Deforestation and the structure of frog communities in the Humedale Terraba-Sierpe, Costa Rica. Zoological Science 26: 197-202.

Gardner, T. A., M. A. Ribeiro-Júnior, J. Barlow, T. Cristina, S. Ávila-Pires, M. S. Hoogmoed & C. A. Peres (2007): The value of primary, secondary, and plantation forests for a neotropical herpetofauna. Conservation Biology 21: 775-787.

Gascon, C. (1993): Breeding-habitat use by five Amazonian frogs at forest edge. Biodiversity and Conservation 2: 438-444.

Germano, J. M., J. M. Sander, R. W. Henderson & R. Powell (2003): Herpetofaunal communities in Grenada: a comparison of altered sites, with an annotated checklist of Grenadian amphibians and reptiles. Caribbian Journal of Science 39: 68-76.

Gillespie, G., S. Howard, D. Lockle, M. Scroggie & Boedl (2005): Herpetofaunal richness and community structure of offshore islands of Sulawesi, Indonesia. Biotropica 37: 279-290.

Gillespie, G. R., E. Ahmad, B. Elahan, A. Evans, M. Ancrenaz, B. Goossens & M. P. Scroggie (2012): Conservation of amphibians in Borneo: relative value of secondary tropical forest and non-forest habitats. Biological Conservation 152: 136-144.

Heinen, J. T. (1992): Comparison of the leaf litter herpetofauna in abandoned cacao plantations and primary rain forest in Costa Rica: some implications for faunal restoration. Biotropica 24: 431-439. Hilje, B. & T. Mitchell Aide (2012): Recovery of amphibian species richness and composition in a consequence of secondary forests, northeastern Costa Rica. Biological Conservation 146: 170-176.

Hillers, A., M. Veith & M.-O. Rödel (2008): Effects of forest fragmentation and habitat degradation on West African leaf-litter frogs. Conservation Biology 22: 762-772.

Hinde, R., G. Corti, E. Fanning & R. K. B. Jenkins (2001): Anurans in the Kilombero Valley, Tanzania: comparison between miombo woodland, evergreen forest and teak plantatios. African Journal of Herpetology 50: 35-39.

Inger, R. F. & R. K. Colwell (1977): Organization of contiguous communities of amphibians and reptiles in Thailand. Ecological Monographs 47: 229-253.

Isaacs Cubides, P. J. & J. N. Urbina Cardona (2011): Anthropogenic disturbance and edge effects on anuran assemblages inhabiting cloud forest fragments in Colombia. Brazilian Journal of Nature Conservation 9: 39-46.

Katwate, U., D. Apte & R. Raut (2013): Diversity and distribution of anurans in Phansad Wildlife Sanctuary (PWS), northern Western Ghats of India. Journal of Threatened Taxa 5: 3589-3602.

Krishna, S. N., S. B. Krishna & K. K. Vijayalaxmi (2005): Variation in anuran abundance along the streams of the Western Ghats, India. Herpetological Journal 15: 167-172.

Krishnamurthy, S. V. (2003): Amphibian assemblages in undisturbed and disturbed areas of Kudremukh National Park, central Western Ghats, India. Environmental Conservation 30: 274-282.

Kudavidangage, E. P., T. C. Wanger, C. de Alwis, S. Sanjeewa & S. W. Kotagama (2011): Amphibian and butterfly diversity across a tropical land-use gradient in Sri Lanka; implications for conservation decision making. Animal Conservation 15: 253-265.

Lieberman, S. S. (1986): Ecology of the leaf litter herpetofauna of a neotropical rain forest: La Selva, Costa Rica. Acta Zoologica Mexicana 15: 1-74.

Malzona, P. K. & M. Veith (2012): Amphibian community along elevational and habitat distrubance gradients in the Taita Hills, Kenya. Herpetotorpicos 7: 7-16.

Maritz, B. (2007): The distribution and abundance of herpetofauna on a quaternary aeolian dune deposit: implications for strip mining. University of the Witwatersrand, Johannesburg.

Murali, R. & T. R. S. Raman (2012): Streamside amphibian communities in plantations and a rainforest fragment in the Anamalai hills, India. Journal of Threatened Taxa 4: 2849-2856.

Murrieta-Galindo, R., A. González-Romero, F. López-Barrera & G. Parra-Olea (2013): Coffee agrosystems: an important refuge for amphibians in central Veracruz, Mexico. Agroforestry Systems 87: 1-13.

Nath, N., S. Sutradhar, A. K. Mani, V. Vijyan, K. Kumar, B. L. Narayana, B. Naresh, G. Baburao, S. Dharwadkar, G. SKrishnan, B. Vinoth, R. Maniraj, D. M. Reddy, D. Adi mallaiah & K. Swamy (2012): Herpetofaunal assemblage with special emphasis on community structure and spatiality in amphibians of Cauvery delta region, Tamil Nadu. Asian Journal of Conservation Biology 1: 78-85.

Ofori-Boateng, C., W. Oduro, A. Hillers, K. Norris, S. K. Oppong, G. B. Adum & M.-O. Rödel (2012): Differences in the effects of selective logging on amphibian assemblages in three West African forest types. Biotropica 45: 94-101.

Parris, K. M. & D. B. Lindenmayer (2004): Evidence that creation of a *Pinus radiata* plantation in south-eastern Australia has reduced habitat for frogs. Acta Oecologica 25: 93-101.

Pawar, S. (1999): Effect of habitat alteration on herpetofaunal assemblages of evergreen forest in Mizoram, Nort-East India. Saurashtra University, Rajkot.

Pawar, S. , G. S. Rawat & B. C. Choudhury (2004): Recovery of frog and lizard communities following primary habitat alteration in Mizoram, Northeast India. BMC Ecology 4: 10.

Pineda, E. & G. Halffter (2004): Species diversity and habitat fragmentation: frogs in a tropical montane landscape in Mexico. Biological Conservation 117: 499-508.

Pineda, E., C. Moreno, F. Escobar & G. Halffter (2005): Frog, bat, and dung beetle diversity in the cloud forest and coffee agroecosystems of Veracruz, Mexico. Conservation Biology 19: 400-410.

Reichert, M. (2004): A comparison of anuran species richness between primary and secondary forest in São Francisco do Pará. Independent Study Project (ISP) Collection 524.

Ribeiro-Júnior, M. A., T. A. Gardner & T. C. S. Ávila-Pires (2008): Evaluating the effectiveness of herpetofaunal sampling techniques across a gradient of habitat change in a tropical forest landscape. Journal of Herpetology 42: 733-749.

Russel, C. & C. T. Downs (2012): Effect of land use on anuran species composion in north-eastern KwaZulu-Natal, South Africa. Applied Geography 35: 247-256.

Santos-Barrera, G. & J. N. Urbina-Cardona (2011): The role of the matrix-edge dynamics of amphibian conservation in tropical montane fragmented landscapes. Revista mexicana de biodiversidad 82: 679-687.

Schlaepfer, M. A. & T. A. Gavin (2001): Edge effects on lizards and frogs in tropical forest fragments. Conservation Biology 15: 1079-1090.

Tocher, M. D., C. Gascon & J. Meyer (2001): Community composition and breeding success of Amazonian frogs in continuous forest and matrix habitat aquatic sites. pp. 235-247 in R. O. J. Bierregaard, C. Gascon, T. E. Lovejoy & R. C. G. Mesquita (eds.). Lessons from Amazonia — the ecology and conservation of a fragmented forest. Yale University Press, New Haven & London.

Tocher, M. D., C. Gascon & B. L. Zimmerman (1997): Fragmentation effects on a Central Amazonian frog community: a ten year study. pp 124-137 in W. F. Laurance & R. O. J. Bierregaard (eds.). Tropical forest remnants - ecology, management, and conservation of fragmented landscapes. The University of Chicago Press, Chicago & London.

Toral C., E., P. Feinsinger & M. L. Crump (2002): Frogs and a cloud-forest edge in Ecuador. Conservation Biology 16: 735-744.

Urbina-Cardona, J. N. & M. C. Londoño-M. (2003): Distribución de la comunidad de herpetofauna asociada a cuatro áreas con diferente grado de perturbación en la Isla Gorgona, Pacífico colombiano. Academia Colombiana de Ciencias 27: 105-113.

Urbina-Cardona, J. N., M. Olivares-Pérez & V. H. Reynoso (2006): Herpetofauna diversity and microenvironment correlates across a pasture–edge–interior ecotone in tropical rainforest fragments in the Los Tuxtlas Biosphere Reserve of Veracruz, Mexico. Biological Conservation 132: 61-75.

Vallan, D. (2000): Influence of forest fragmentation on amphibian diversity in the nature reserve of Ambohitantely, highland Madagascar. Biological Conservation 96: 31-43.

Vallan, D. (2002): Effects of anthropogenic environmental changes on amphibian diversityin the rain forests of eastern Madagascar. Journal of Tropical Ecology 18: 725-742.

Vallan, D., F. Andreone, V. H. Raherisoa & R. Dolch (2004): Does selective wood exploitation affect amphibian diversity? The case of An’Ala, a tropical rainforest in eastern Madagascar. Oryx 38: 410-417.

Vonesh, J. R. (2001): Patterns of richness and abundance in a tropical African leaf-litter herpetofauna. Biotropica 33: 502-510.

Wanger, T. C., D. T. Iskandar, I. Motzke, B. W. Brook, N. S. Sodhi, Y. Clough & T. Tscharntke (2011): Effects of landuse change on community composition of tropical amphibians and reptiles in Sulawesi, Indonesia. Conservation Biology 24: 795-802.

Watling, J. I. & M. A. Donnelly (2007): Multivariate correlates of extinction proneness in a naturally fragmented landscape. Diversity and Distribution 13: 372-378.
