# Supplementary material for: What makes a successful species? Traits facilitating survival in altered tropical forests
Source: BMC Ecol. 2017 Jun 28;17:25. doi: 10.1186/s12898-017-0135-y (PMC5490239; doi:10.1186/s12898-017-0135-y)
Supplement: Supplementary file 2 — Additional file 2. Anuran traits references. References to journal articles, books and web resources containing information on species traits for the species included in the primary data set on anuran occurrence. [file 12898_2017_135_MOESM2_ESM.docx]

**Additional file 2. Anuran traits references.**

References to journal articles, books and web resources containing information on species traits for the species included in the primary data set on anuran occurrence.

**Journal articles and Books:**

Abravaya, J. P. & J. F. Jackson (1978): Reproduction in *Macrogenioglottus alipioi* Carvalho (Anura, Leptodactylidae). Serial Publications of the Natural History Museum of Los Angeles County 298: 1-9.

Adler, K. (1965): Three new frogs of the genus Hyla from the Sierra Madre del Sur of Mexico. Occasional Papers of the Museum of Zoology University of Michigan 642: 1-18.

Adum, B. A., C. Ofori-Boateng, W. Oduro & M.-O. Rödel (2011): Re-discovery of the giant West African squeaker, *Arthroleptis krokosua* Ernst, Agyei & Rödel, 2008 (Amphibia: Anura: Arthroleptidae) in two forests of southwestern Ghana with observations on the species’ variability and habitat preferences. Zootaxa 2744: 34-38.

Alcala, A. C. & W. C. Brown (1998): Philippine amphibians: an illustrated field guide. Bookmark, Makati City, Philippines.

Amiet, J.-L. (1977): Les *Astylosternus* du Cameroun (Amphibia anura, Astylosterninae). Annales de la Faculté des Science de Yaoundé 23-24: 99-237.

Amiet, J.-L. (1991): Images d’amphibiens camerounais. III. Le comportement de garde des oeufs Alytes 9: 15-22.

Amiet, J.-L. (2012). Les rainettes du Cameroun. J.-L. Amiet and La Naf des Livre, Sain-Nazaire.

Anahi Güizado-Rodriguez, M., U. Omar Garcia-Vazquez & J. L. Aguilar-Lopez (2010): Thermoregulation of *Craugastor berkenbuschii* (Peters, 1870). Herpetological bulletin 112: 4-6.

Andreone, F. & R. A. Nussbaum (2006): A revision of *Mantidactylus microtis* and *M. microtympanum*, and a comparison with other large Madagascan stream frogs (Anura: Mantellidae: Mantellinae). Zootaxa 1105: 49-68.

Andreone, F., G. M. Rosa & A. P. Raselimanana (2014). Les amphibiens des zones arides de l’ouest et du sud de Madagascar. Association Vahatra, Antananarivo.

Angel, F. (1940): Description de trois amphibiens nouveaux du Cameroun, matéraux de la mission *P. lepesme*, *R. paulian* et *A. villiers* (2e note). Bulletin du Muséum national d’histoire naturelle Paris 12: 238-243.

Anstis, M. (2013): Tadpoles and frogs of Australia. New Holland, Sindney.

Ao, M., S. Bordoloi, A. Ohler & S. Grosjean (2006): *Rana khare* (Kiyasetuo & Khare, 1986): present distribution, redescription of holotype and morphology of adults and tadpoles. Alytes 24: 22-39.

Ariyasiri, K., G. Bowatte, U. Menike, S. Meegaskumbura & M. Meegaskumbura (2011): Predator-induced plasticity in tadpoles of *Polypedates cruciger* (Anura: Rhacophoridae). Amphibian and Reptile Conservation 5: 14-21.

Artenga, A., L. Bustamante & J. M. Guayasamin (2014): The amphibians and reptiles of Mindo — life in the cloudforest. Universidad Tecnológica Indoamérica, Quito.

Ávila, R. W., A. Pansonato & C. Strüssmann (2010): A new species of the *Rhinella margaritifera* group (Anura: Bufonidae) from Brazilian Pantanal. Zootaxa 2339: 57-68.

Bahir, M. M., M. Meegaskumbura, K. Manamendra-Arachchi, C. J. Schneider & R. Pethiyagoda (2005): Reproduction and terrestrial direct development in Sri Lankan shrub frogs (Ranidae: Rhacophorinae: *Philautus*). The Raffles Bulletin of Zoology 12: 339-350.

Barej, M. F., M.-O. Rödel, N. L. Gonwouo, O. S. G. Pauwels, W. Böhme, & A. Schmitz (2010): Review of the genus *Petropedetes* Reichenow, 1874 in Central Africa with the description of three new species (Amphibia: Anura: Petropedetidae). Zootaxa 2340: 1-49.

Barej, M. F., A. Schmitz, M. Menegon, A. Hillers, H. Hinkel, W. Böhme & M.-O. Rödel (2011): Dusted off — the African *Amietophrynus superciliaris*-species complex of giant toads. Zootaxa 2772: 1-32.

Barreto, L. & G. V. Andrade (1995): Aspects of the reproductive biology of *Physalaemus cuvieri* (Anura: Leptodactylidae) in northeastern Brazil. Amphibia-Reptilia 16: 67-76.

Barrio-Amorós, C. L., J. M. Guayasamin & S. B. Hedges (2012): A new minute Andean *Pristimantis* (Anura: Strabomantidae) from Venezuela. Phyllomedusa 11: 83-93.

Barrio-Amorós, C. L. & H. Kaiser (2008): Distribution of Strabomantis biporcatus (Terrarana: Strabomantidae) in northern Venezuela, with comments on its phenotypic variation. Salamandra 44: 248-254.

Barros, F. B., L. Vicente & H. M. Pereira (2010): Amphibians of the Riozinho do Anfrísio Extractive Reserve, Pará. Rapid Color Guide.

Bennett, D., K. Hampson, K. Sanders & M. Anderson (1998): Frogs of Coorg, Karnataka, India. Aberdeen University Western Ghats Project 1998.

Berry, P. Y. & J. R. Hendrickson (1963): Leptobrachium nigrops, a new pelobatid frog from the Malay Peninsula, with remarks on the genus Leptobrachium in southeastern Asia. Copeia 1963: 643-648.

Biju, S. D. (2003): Reproductive mode in the shrub frog *Philautus glandulosus* (Jerdon, 1853) (Anura: Rhacophoridae). Current Science 84: 283-284.

Biju, S. D. & F. Bossuyt (2009): Systematics and phylogeny of *Philautus* Gistel, 1848 (Anura, Rhacophoridae) in the Western Ghats of India, with descriptions of 12 new species. Zoological Journal of the Linnean Society 155: 374-444.

Biju, S. D., I. van Bocxlaer, V. B. Giri, S. P. Loader & F. Bossuyt (2009): Two new endemic genera and a new species of toad (Anura: Bufonidae) from the Western Ghats of India. BMC research notes 2: 241.

Biju, S. D., I. van Bocxlaer, S. Mahony, K. P. Dinesh, C. Radhahrishnan, A. Zachariah, V. Giri & F. Bossuyt (2011): A taxonomic review of the night frog genus *Nyctibatrachus* Boulenger, 1882 in the Western Ghats, India (Anura: Nyctibatrachidae) with description of twelve new species. Zootaxa 3029: 1-96.

Bogert, C. M. (1969) The eggs and hatchlings of the Mexican leptodactylid frog *Eleutherodactylus decoratus* Taylor. American Museum novitates 2376: 1-9.

Bolívar-G, W., A. Giraldo & J. Mendez (2011): Amphibia, Anura, Strabomantidae, *Pristimantis palmeri* Boulenger, 1912: distribution extension for the Central Cordillera, Colombia. CheckList 7: 9-10.

Bopage, M. M., K. Wewalwala, M. Krvavac, O. Jovanovic, G. Safarek & V. Pushpamal (2011): Species diversity and threat status of amphibians in the Kanneliya Forest, lowland Sri Lanka. Salamandra 47: 173-177.

Boulenger, G. A. (1896): II. — Descriptions of new Reptiles and Batrachians from Colombia. Journal of Natural History 17: 16-21.

Boulenger, G. A. (1898): Fourth report on additions to the batrachian collection in the Natura-History Museum Proceedings of the Zoological Society of London 66: 473-482.

Boulenger, G. A. (1920): A monograph of the South Asian, Papuan, Melanesian and Australian frogs of the genus *Rana*. Zoological Survey of India, Calcutta.

Bourne, G. R. (1997): Reproductive behaviour of terrestrial breeding frogs *Eleutherodactylus johnstonei* in Guyana. Journal of Herpetology 31: 221-229.

Brauer, K. (1991): Kröten. Urania, Leipzig. Buchacher, C. (1993): Field studies on the Small Surinam toad *Pipa arrabali* near Manaus, Brazil. Institut für Zoologie der Universität Wien, Wien.

Caldwell, J. P. (1991). A new specis of toad in the genus *Bufo* from Pará, Brazil, with an unusual breeding site. Papéis Avulsos de Zoologia 37: 389-400.

Caldwell, J. P. & P. T. Lopez (1989): Foam-generating behavior in tadpoles of Leptodactylus mystaceus. Copeia 1989: 498-502.

Campbell, J. A., J. C. Blancas-Hernández & E. N. Smith (2009): A new species of stream-breeding treefrog of the genus *Charadrahyla* (Hylidae) from the Sierra Madre del Sur of Guerrero, Mexico. Copeia 2009: 287-295.

Canedo, C., M. Dixo & J. P. Pombal Jr. (2004): A new species of *Chiasmocleis* Méhelÿ, 1904 (Anura, Microhylidae) from the Atlantic rainforest of Bahia, Brazil. Herpetologica 60: 495-501.

Cannatella, D. C. (1986): A new species of *Osornophryne* (Anura: Bufonidae) from the Andes of Ecuador. Copeia 1986. Caramaschi, U. & C. A. G. da Cruz (1997): Redescription of

*Chiasmocleis albopunctata* (Boettger) and description of a new species of Chiasmocleis (Anura: Microhylidae). Herpetologica 53 : 259-268.

Caramaschi, U. & J. P. Pombal Jr. (2006): A new species of *Rhinella Fitzinger*, 1826 from the Atlantic Rain Forest, Eastern Brazil (Amphibia, Anura, Bufonidae). Papéis Avulsos de Zoologia 46: 251-259.

Cardozo, D. & F. Lobo (2009): *Pseudopaludicola mirandae* Mercadal de Barrio and Barrio, 1994 (Anura, Leiuperidae) is a junior synonym of *Pseudopaludicola boliviana* Parker, 1927. Journal of Herpetology 43: 685-687.

Carruthers, V. (2001): Frogs and frogging in southern Africa. Struik Publishers, Cape Town.

Chanda, S. K. (2002): Handbook. Indian Amphibians. Zoological Survey of India, Calcutta.

Chandramouli, S. R. & S. R. Ganesh (2010): Herpetofauna of Southern Western Ghats, India — reinvestigated after decades. Taprobanica 2: 72-85.

Chandramouli, S. R., S. R. Ganesh & N. Baskaran (2011a). On recent sightings of a little-known south Indian toad, *Duttaphrynus hololius* (Günther, 1876) with notes on its morphological characterization and ecology. Herpetology Notes 4: 271-274.

Chandramouli, S. R., S. Harikrishnan S. & K. Vasudevan (2011b): Little known endemic frogs of the Andaman Islands. FrogLog 98: 16-17.

Chandramouli, S. R. & A. Kalaimani (2014): Description of the larvae of Günther’s toad *Duttaphrynus hololius* (Günther, 1876) (Anura: Bufonidae) with notes on development and oral ultra-structure. Alytes 31: 3-12.

Channing, A. (2001): Amphibians of Central and Southern Africa. Cornell University Press, Ithaca.

Channing, A. & K. M. Howell (2006): Amphibians of East Africa. Cornell University Press, Ithaca.

Channing, A., M.-O. Rödel & J. Channing (2012): Tadpoles of Africa. The biology and identification of all known tadpoles in sub-Saharan Africa. Edition Chimaira, Frankfurt/M.

Chávez, G., C. H. Cosmópolis & L. Luján (2013): Annotated checklist and ecological notes of anurans from the Southern region of Yanachaga Chemillen National Park, Central Andes of Peru. Herpetotropics 8: 23-18.

Chochran, D. M. (1927): New reptiles and batrachians collected by Dr. Hugh M. Smithn Siam. Proceedings of the Biological Society of Washington 40: 179-192.

Cochran, D. M. & C. J. Coin (1970): Frogs of Colombia. Smithsonian Institution Press, Washington.

Coe, M. J. (1967): Co-operation of three males in nest construction by *Chiromantis rufescens* Günther (Amphibia: Rhacophoridae). Nature 214: 112-113.

Crump, M. L. (1974): Reproductive strategies in a tropical anuran community. University of Kansas Museum of Natural History Miscellaneous Publications 61: 1-68.

da Cruz, C. A. G., U. Caramaschi & E. Izecksohn (1997): The genus Chiasmocleis Méhely, 1904 (Anura, Microhylidae) in the Atlantic Rain Forest of Brazil, with description of three new species. Alytes 15: 49-71.

da Cruz, C. A. G. & M. F. Napoli (2010): A new species of smooth horned frog, genus Proceratophrys Miranda-Ribeiro (Amphibia: Anura: Cycloramphidae), from the Atlantic Rainforest of eastern Bahia, Brazil. Zootaxa 2660: 57-67.

Daniels, R. J. R. (2005): Amphibians of peninsular India. Indian Academy of Science & University Press, Hyderabad.

Das, I. (2007): The amphibians and reptiles of Brunei. A pocket guide. Natural History Publications (Borneo), Koto Kinabalu.

Das, I. & S. K. Dutta (2006): New species of *Polypedates* (Anura: Rhacophoridae) from the Western Ghats, Southwest India. Journal of Herpetology 40: 214-220.

Das, I. & A. Haas (2010): New species of *Microhyla* from Sarawak: Old World’s smallest frogs crawl out of miniature pitcher plants on Borneo (Amphibia: Anura: Microhylidae). Zootaxa 2571: 37-52.

Das, I., A. Jankowski, M. I. B. Makmor & A. Haas (2007): Species diversity, elevational distribution and reproductive modes in an amphibian community at the Matang Range, Sarawak (Borneo). Mitteilungen des Hamburger Zoologischen Museums und Instituts 104: 141-171.

de Almeida Prado, C., L. F. Toledo, J. Zina & C. F. B. Haddad (2005): Trophic eggs in the foam nests of *Leptodactylus labyrinthicus* (Anura, Leptodactylidae): an experimental approach. The Herpetological Journal 15: 279-284.

de Almeida Prado, C. P., M. Uetanabaro, C. F. B. Haddad & C. Guyer (2002): Description of a new reproductive mode in *Leptodactylus* (Anura, Leptodactylidae), with a review of the reproductive specialization toward terrestriality in the genus. Copeia 2002: 1128-1133.

de Almeida Prado, C. P., M. Uetanabaro & F. S. Lopes (2000): Reproductive strategies of *Leptodactylus chaquensis* and *L. podicipinus* in the Pantanal, Brazil. Journal of Herpetology 34: 135-139.

de Carvalho, A. L. (1946): Um novo género de Ceratofridideo do sudeste Baiano. Boletim do Museu Nacional. Zoologia 73: 1-19.

de la Riva, I. (1999): A new *Phyllomedusa* from southwestern Amazonia (Amphibia: Anura: Hylidae). Revista espanola de herpetología 13: 123-131.

de Mattos Brito, L. B., L. Bezerra, F. Aguiar, C. Moura-Neto, C. A. Zucco & P. Cascon (2013): Diet, activity patterns, microhabitat use and defensive strategies of *Rhinella hoogmoedi* Caramaschi & Pombal, 2006 from a humid forest in northeast Brazil. The Herpetological Journal 23: 29-37.

de Oliveira Filho, J. C., H. C. de Moura Costa & Ú. M. L. Braga (2005): Egg-laying and foam-beating in *Leptodactylus fuscus* (Anura, Leptodactylidae). Biota Neotropica 5: 1-2.

de Oliveira Filho, J. C. & A. A. Giaretta (2008): Reproductive behavior of *Leptodactylus mystacinus* (Anura, Leptodactylidae) with notes on courtship call of other *Leptodactylus* species. Iheringia. Série Zoologia 98: 508515.

Dehling, J. M. & D. M. Dehling (2013) Range extension of *Rhacophorus dulitensis* Boulenger, 1892 (Amphibia: Anura: Rhacophoridae) in western Borneo. Check List 9: 425-426.

Deichmann, J. L., W. E. Duellman & B. G. Williamson (2008): Predicting biomass from snout-vent length in New World frogs. Journal of Herpetology 42: 238-245.

Dixo, M. (2004): Range extension of *Rhacophorus dulitensis* Boulenger, 1892 (Amphibia: Anura: Rhacophoridae) in western Borneo. Phyllomedusa 3: 77-79.

Downie, J. R. (1996): A new example of female parental bahaviour in *Leptodactylus validus*, a frog of the *Leptodactylid melanotus’* species group. Herpetological Journal 6: 32-34.

du Preez, L. & V. Carruthers (2007): Complete Guide to the Frogs of Southern Africa. Struik Publishers, Cape Town.

Duellman, W. E. (1960): Synonymy, variation, and distribution of *Ptychohyla leonhard-schultzei* Ahl. Studies of American Hylid frogs. IV. Herpetologica 16: 191-197.

Duellman, W. E. (2001): Hylid Frogs of Middle America (Contribution to Herpetology). 2 edition. Society for the study of amphibians and reptiles, Ithaca, New York.

Duellman, W. E. (2005): Cusco Amazónico — the lives of amphibians and reptiles in an Amazonian rainforest. Comstock Publishing Associates, Ithaca.

Duellman, W. E. & M. L. Crump (1974): Speciation in frogs of the *Hyla parviceps* group in the upper Amazon Basin. Occasional Papers of the Museum of Natural History, The University of Kansas 23: 1-40.

Duellman, W. E. & E. Lehr. (2009): Terrestrial-breeding frogs (Strabomantidae) in Peru. Natur & Tier Verlag, Münster.

Duellman, W. E. & J. Lescure (1973): Life history and ecology of the Hylid frog *Osteocephalus taurinus*, with observations on larval behavior. Occasional Papers of the Museum of Natural History, The University of Kansas 13: 1-12.

Duellman, W. E. & M. Lizana (1994): Biology of a sit-and-wait predator, the Leptodactylid frog *Ceratophrys cornuta*. Herpetologica 50: 51-64.

Duellmann, W. E. (1978): The biology of an equatorial herpetofauna in Amazonian Ecauador. Miscellaneous Publication, Museum of Natural History, University of Kansas 65: 1-352.

Ernst, R., A. C. Agyei & M.-O. Rödel (2008): A new giant species of *Arthroleptis* (Amphibia: Anura: Arthroleptidae) from the Krokosua Hills Forest Reserve, south-western Ghana. Zootaxa 1697: 58-68.

Fabrezi, M., S. I. Quinzio & J. Goldberg (2009): Giant tadpole and delayed metamorphosis of *Pseudis platensis* Gallardo, 1961 (Anura, Hylidae). Journal of Herpetology 43: 228-243.

Fernando, P. & N. Dayawansa (1995): Description of the larval stages and notes on the reproduction of *Polypedates longinasus* (Ahl, 1927) (Amphibia, Rhacophoridae). Journal South Asian Natural History 1: 235-240.

Fernando, P., N. Dayawansa & M. Siriwardena (1994): Bufo kotagamai, a new toad (Bufonidae) from Sri Lanka. Journal of South Asian natural History 1: 119-124.

Firschein, I. L. (1950): A new toad from Mexico with a redefinition of the cristatus group. Copeia 1950: 81-87.

Frétey, T., M. Dewynter & C. P. Blanc (2011): Amphibiens d’Afrique centrale et d’Angola. Clé de détermination Illustrée des Amphibiens du Gabon et du Mbini. Biotope, Mèze (Collection Parthénope) & Muséum National d’Histoire Naturelle, Paris.

Garda, A. A., G. M. Biavati & G. C. Costa (2006): Sexual dimorphism, female fertility, and diet of Pipa arrabali (Anura, Pipidae) in Serra do Cachimbo, Pará, Brazil. South American Journal of Herpetology 1: 20-24.

Garda, A. A., D. J. Santana & V. A. de Avelar São-Pedro (2010): Taxonomic characterization of paradoxical frogs (Anura, Hylidae, Pseudae): geographic distribution, external morphology, and morphometry. Zootaxa 2666: 1-28.

Gaulke, M. (2011): The herpetofauna of Panay Island, Philippines. Edition Chimaira, Frankfurt/M.

Gawor, A., K. van der Straeten, D. Karbe, U. Manthey & T. Ziegler (2011): Reproduction and development of the dark-sided frog *Hylarana nigrovittata* sensu lato at the Cologne Zoo. Salamandra 47: 1-8.

Giaretta, A. A. & M. N. de C. Kokubum (2004): Reproductive ecology of *Leptodactylus furnarius* Sazima & Bokermann, 1978, a frog that lays eggs in underground chambers. Herpetozoa 16: 115-126.

Giaretta, A. A. & K. G. Facure (2006): Terrestrial and communal nesting in *Eupemphix nattereri* (Anura, Leiuperidae): interactions with predators and pond structure. Journal of Natural History 40: 2577-2587.

Giaretta, A. A. & K. G. Facure (2009): Habitat, egg-laying behaviour, eggs and tadpoles of four sympatric species of *Pseudopaludicola* (Anura, Leiuperidae). Journal of Natural History 43: 995-1009.

Gillespie, G. R., M. Anstis, S. D. Howard & D. Lockie (2007): Description of the tadpole of the Rhacophorid frog *Rhacophorus georgii* Roux (Rhacophoridae) from Sulawesi, Indonesia. Journal of Herpetology 41: 150-153.

Gillespie, G. R., D. Lockie, M. P. Scroggi & D. T. Iskandar (2004) Habitat use by stream-breeding frogs in southeast Sulawesi, with some preliminary observations on community organization. Journal of Tropical Ecology 20: 439-448.

Girish, S. & S. K. Saidapur (2003): Density-dependent growth and metamorphosis in the larval bronze frog *Rana temporalis* is influenced by genetic relatedness of the cohort. Journal of biosciences 28: 489-496.

Glaw, F. & M. Vences (1992): Zur Kenntnis der Gattungen *Boophis*, *Aglyptodactylus* und *Mantidactylus* (Amphibia: Anura) aus Madagaskar, mit Beschreibung einer neuen Art. Bonner Zoologische Beiträge 43: 45-77.

Glaw, F. & M. Vences (1997): Neue Ergebnisse zur *Boophis goudoti*-Gruppe aus Madagaskar: Bioakustik, Fortpflanzungsstrategien und Beschreibung von *Boophis rufioculis* sp. nov. Salamandra 32: 225-242.

Glaw, F. & M. Vences (1999): Resurrection and redescription of *Mantidactylus tricinctus* from eastern Madagascar. Journal of Herpetology 33: 639-647.

Glaw, F. & M. Vences (2002): A new cryptic frog species of the *Mantidactylus boulengeri* group with a divergent vocal sac structure. Amphibia-Reptilia 23: 293-304.

Glaw, F. & M. Vences (2004): A preliminary review of cryptic diversity in frogs of the subgenus *Ochthomantis* based on mtDNA sequences and morphology (Anura, Mantellidae, *Mantidactylus*). Spixiana 27: 83-91.

Glaw, F. & M. Vence. (2007): A field guide to the amphibians and reptiles of Madagascar. 3 edition. Vences & Glaw Verlags GbR.

Glaw, F., M. Vences & V. Gossmann (2000): A new species of Mantidactylus (subgenus *Guibemantis*) fromMadagascar, with a comparative survey of internal femoral gland structure in the genus (Amphibia: Ranidae: Mantellinae). Journal of Natural History 34: 1135-1154.

Gomez-Mestre, I., R. A. Pyron & J. J. Wiens (2012): Phylogenetic analyses reveal unexpected patterns in the evolution of reproductive modes in frogs. Evolution 66: 3687-3700.

Gopalan, S. V., A. Nair, K. S. Kumar, J. Merilä & S. George (2012): Morphology of *Indirana semipalmata* (Boulenger, 1882) (Amphibia; Anura) adults and tadpoles from the Western Ghats, India. Herpetology Notes 5: 263-273.

Goris, R. C. & N. Maeda (2004): Guide to the amphibians and reptiles of Japan. Krieger Publishing Company, Malabar.

Grismer, L. L. (2011): Amphibians and reptiles of the Seribuat Archipelago. Edition Chimaira, Frankfurt/M.

Grismer, L. L., T. Neang, T. Chav, P. L. Wood Jr, J. R. Oaks, J. Holden, J. L. Grismer, T. R. Szutz & T. M.

Youmans (2008): Additional amphibians and reptiles from the Phnom Samkos Wildlife Sanctuary in northwestern Cardamom Mountains, Cambodia, with comments on their taxonomy and the discovery of three new species. The Raffles Bulletin of Zoology 56: 161-175.

Grosjean, S., A. Strauß, J. Glos, R.-D. Randrianiaina, A. Ohler & M. Vences (2011): Morphological and ecological uniformity in the funnel-mouthed tadpoles of Malagasy litter frogs, subgenus *Chonomantis*. Zoological Journal of the Linnean Society 162: 149-183.

Grosjean, S., M. Thomas, F. Glaw & M. Vences (2006): The tadpole of the Malagasy treefrog *Boophis rufioculis*: molecular identification and description. Spixiana 29: 73-76.

Guimarães, T. C. S., G. B. de Figueiredo, D. O. Mesquita & M. M. Vasconcellos (2011): Ecology of *Hypsiboas albopunctatus* (Anura: Hylidae) in a neotropical savanna. Journal of Herpetology 45: 244-250.

Gururaja, K. V. (2010a): Novel reproductive mode in a torrent frog *Micrixalus saxicola* (Jerdon) from the Western Ghats, India. Zootaxa 2642: 45-52.

Gururaja, K. V. (2010b): Pictorial guide to frogs and toads of the Western Ghats. Sharadh Enterprises, Karnataka.

Haas, A. & I. Das (2008): Larval identities of *Ansonia hanitschi* Inger, 1960 (Amphibia: Bufonidae) and *Polypedates colletti* (Boulenger, 1890) (Amphibia: Rhacophoridae) from East Malaysia (Borneo). Salamandra 44: 85-100.

Haddad, C. F. B., L. F. Toledo, C. P. A. Prado, D. Loebmann, J. L. Gasparini & I. Sazima (2013): Guia dos Anfíbios da Mata Atlântica: Diversidade e Biologia. Anolisbooks, Sao Paulo.

Hailey, A. (ed.) (2011): OGATT: The online guide to the animals of Trinidad and Tobago. Department of Life Sciences, The University of the West Indies, St. Augustine.

Hamidy, A. & M. Matsui (2010): A new species of blue-eyed *Leptobrachium* (Anura: Megophryidae) from Sumatra, Indonesia. Zootaxa 2395: 34-44.

Harper, E. B., G. J. Measey, D. A. Patrick, M. Menegon & J. R. Vonesh (2010): Field Guide to the Amphibians of the Eastern Arc Mountains and Coastal Forests of Tanzania and Kenya. Camerapix Publishers International, Nairobi.

Hartmann, T., F. Ihlow, S. Edwards, S. Sothanin, M. Handschuh & W. Böhme (2013): A preliminary annotated checklist of the amphibians and reptiles of the Kulen Promtep Wildlife Sanctuary in Northern Cambodia. Asian Herpetological Research 4: 36-55.

Herrmann, H.-W., W. Böhme, P. A. Herrmann, M. Plath, A. Schmitz & M. Solbach (2005): African biodiversity hotspots: the amphibians of Mt. Nlonako, Cameroon. Salamandra 41: 61-81.

Heyer, W. R. (1973): Ecological interactins of frog larvae at a seasonal tropical location in Thailand. Journal of Herpetology 7: 337-361.

Heyer, W. R. (1978): Systematics of the fuscus group of the frog genus Leptodactylus (Amphibia, Leptodactylidae). Science Bulletin. Natural History Museum of Los Angeles County 29: 1-85.

Heyer, W. R. (1983): Clarification of the names *Rana mysacea* Spix, 1824, *Leptodactylus amazonicus* Heyer, 1978 and a description of a new species, *Leptodacylus spixi* (Amphibia: Leptodactylidae). Proceedings of the Biological Society of Washington 96: 270-272.

Heyer, W. R. (1988): A notable collection of *Cycloramphus* (Amphibia: Leptodactylidae) from Bahia, Brazil, with a description of a new species (*Cycloramphus migueli*). Proceedings of the Biological Society of Washington 101: 151-154.

Heyer, W. R. (1994): Variation within the *Leptodactylus podicipinus-wagneri* complex of frogs (Amphbia: Leptodactylidae). Smithonian Contributions to Zoology 546: 1-124.

Heyer, W. R. & M. S. Bellin (1973): Ecological notes on five sympatric *Leptodactylus* (Amphibia, Leptodactylidae) from Ecuador. Herpetologica 29: 66-72.

Heyer, W. R. & A. A. Giaretta (2009): Advertisement calls, notes on natural history, and distribution of *Leptodactylus chaquensis* (Amphibia: Anura: Leptodactylidae) in Brasil. Proceedings of the Biological Society of Washington 122: 292-305.

Heyer, W. R. & M. M. Heyer (2002): *Leptodactylus elenae* Heyer. Catalogue of American Amphibians and Reptiles 742: 1-5.

Heyer, W. R. & M. M. Heyer (2006): *Leptodactylus knudseni* Heyer. Knudsen’s thin-toed frog. Catalogue of American Amphibians and Reptiles 807: 1-12.

Heyer, W. R. & M. M. Heyer (2012): Systematics, distribution, and bibliography of the frog *Leptodactylus validus* (Amphibia: Leptodactylidae). Proceedings of the Biological Society of Washington 125: 276-294.

Heyer, W. R., M. M. Heyer & R. O. de Sá (2010): *Leptodactylus savagei* Heyer. Savage’s thin-toed frog. Catalogue of American Amphibians and Reptiles 867: 1-19.

Heyer, M. M., W. R. Heyer, S. Spear & R. O. de Sá (2003): *Leptodactylus mystacinus* (Burmeister), Mustached Frog. Catalogue of American Amphibians and Reptiles 767: 1-11.

Heyer, W. R. & A. J. Wolf (1989): *Physalaemus crombiei* (Amphibia, Leptodactylidae), a new frog species from Espirito-Santo, Brazil with comments on the *Physalaemus signifer* group. Proceedings of the Biological Society of Washington 102: 500-506.

Hirschfeld, M., M. F. Barej, S. P. Loader & M.-O. Rödel (2012): Description of two *Werneria* tadpoles from Cameroon (Amphibia: Anura: Bufonidae). Zootaxa 3172: 65-68.

Hödl, W. (1990): Reproductive diversity in Amazonian lowland frogs. pp 41-60 in W. Hanke (ed.). Biology and physiology of amphibians. Gustav Fischer Verlag, Stuttgart, New York.

Hoffmann, P. (1995): Zur Kenntniss des Engmaulfrosches *Chaperina fusca* Moquaard, 1892 vom Mt. Kinabalu auf Borneo. Herpetofauna 17: 27-29.

Hoogmoed, M., D. Borges & P. Cascon (1994): Three new species of the genus *Adelophryne* (Amphibia: Anura: Leptodactylidae) from northeastern Brazil, with remarks on the other species of the genus. Zoologische Mededelingen 68: 271-300.

Inger, R. F., H. B. Shaffer, M. Koshy & R. Bakde (1984): A report on a collection of amphibians and reptiles from the Ponmudi, Kerala, South India. The Journal of the Bombay Natural History Society 81: 551-570.

Inger, R. F., B. L. Stuart & D. T. Iskandar (2009): Systematics of a widespread Southeast Asian frog, *Rana chalconota* (Amphibia: Anura: Ranidae). Zoological Journal of the Linnean Society 155: 123-147.

Inger, R. F. & R. B. Stuebing (1997): A field guide to the frogs of Borneo. 2 edition. Natural History Publications (Borneo), Koto Kinabalu.

Iskandar, D. T., B. J. Evans & J. A. McGuire (2014): A novel reproductive mode in frogs: a new species of fanged frog with internal fertilization and birth of tadpoles. PLoS ONE 9: e115884.

Jansen, M., J. Q. Vidoz & O. Helmig (2008): *Physalaemus cuvieri*. Herpetological Review 39: 106-107.

Jones, C. (1971): Notes on hairy frogs (*Trichobatrachus robustus* Boulenger) collected in Rio Muni, West Africa. Herpetologica 27: 51-54.

Jungfer, K.-H. & W. Hödl (2002): A new species of *Osteocephalus* from Ecuador and a redescription of *O. leprieurii* (Duméril & Bibron, 1841) (Anura: Hylidae). Amphibia-Reptilia 23: 21-46.

Jungfer, K.-H. & P. Weygoldt (1999): Biparental care in the tadpole-feeding Amazonian treefrog *Osteocephalus oophagus*. Amphibia-Reptilia 20: 235-249.

Kabir, M., K. Hasa & M. Ahmed (2010): *Chiromantis vittatus* recorded for the first time in Bangladesh. frog leg 14: 2-3.

Kadadevaru, G. G. & R. D. Knamandi (2000): Courtship and nesting behaviour of the Malabar gliding frog *Rhacophorus malabaricus* (Jerdon, 1870). Current Science 79: 377-380.

Kaplan, M. & P. Heimes (2011): Tadpole of *Plectrohyla arborescandens*, with comments on the identity of the tadpole of *Plectrohyla cyclada*. Journal of Herpetology 45: 463-464.

Kaplan, M. & P. M. Ruíz (1997): Two new species of *Hyla* from the Andes of Central Colombia and their relationships to other small Andean Hyla. Journal of Herpetology 31: 230-244.

Karsen, S. J., M. W.-N. Lai & A. Bogadek (1986): Hong Kong amphibians and reptiles. Urban council, Hong Kong.

Kehr, A. I. & V. I. Gómez (2009): Intestinal, body and tail plasticity in *Rhinella schneideri* (Bufonidae) tadpoles induced by a predator insect (Belostoma elegans). Advanced Studies in Biology 1: 85-94.

Knoll, A., J. Köhler, F. Glaw, M. Teschke & M. Vences (2007): Larval morphology in four species of Madagascan frogs of the subgenus Brygoomantis (Mantellidae: Mantidactylus). Zootaxa 1616: 49-59.

Kobel, H. R., L. du Pasquier & H. Gloor (1980): Xenopus amieti sp. nov (Anura: Pipidae) from the Cameroon, another case of tretraploidy. Revue suisse de Zoologie 87: 919-926.

Koch, A., E. Arida, A. Riyanto & W. Böhme (2009): Islands between the realms: a revised checklist of the herpetofauna of the Talaud Archipelago, Indonesia, with discussion about its biogeographic affinities. Bonner Zoologische Beiträge 56: 107-1298.

Köhler, G. (2011): Amphibians of Central America. Herpeton, Offenbach.

Köhler, J. (2000): Amphibian diversity in Bolivia: a study with special reference to montane forest regions. Bonner Zoologische Monographien 48: 1-243.

Köhler, J., M. Vences, N. D’Cruze & F. Glaw (2010): Giant dwarfs: discovery of a radiation of large-bodied ‘stump-toed frogs’ from karstic cave environments of northern Madagascar. Journal of Zoology 282: 21-38.

Kok, P. J. R. & M. Kalamandeen (2008): Introduction to the taxonomy of the amphibians of Kaieteur National Park, Guyana. ABC Taxa 5: 1-278.

Kouamé, N. G. N., B. Tohé, N. G. E. Assemian, G. Gourène & M.-O. Rödel (2008): Prey composition of two syntopic *Phrynobatrachus* species in the swamp forest of Banco National Park, Ivory Coast. Salamandra 44: 177-186.

Krishna, S. N. & S. B. Krishna (2005): Female courtship calls of the litter frog (*Rana curtipes*) in the tropical forests of Western Ghats, South India. Amphibia-Reptilia 26: 431-435.

Krishna, S. N., S. B. Krishna & K. K. Vijayalaxmi (2004): Breeding ecology of a rare microhylid, *Ramanella montana*, in the forests of Western Ghats, India. Current Science 87: 80-82.

Krishnamurthy, S. V., A. H. Manjunatha Reddy & K. V. Gururaja (2001): A new species of frog in the genus *Nyctibatrachus* (Anura: Ranidae) from Western Ghats, India. Current Science 80: 887-891.

Kubicki, B. (2007): Ranas de vidrio — Costa Rica — glass frogs. Instituto Nacional de Biodiversidad, Santo Domingo de Heredia. Lambiris, A. J. L. (1989): The frogs of Zimbabwe. Museo Regionale di Scienze Naturali, Torino.

Laufer, G. & J. M. Barreneche (2008): Re-description of the tadpole of *Pseudopaludicola falcipes* (Anura: Leiuperidae), with comments on larval diversity of the genus. Zootaxa 1760: 50-58.

Lee, J. C. (1996): The amphibians and reptiles of the Yucatan Peninsula. Comstock Publishing Associates., Ithaca, New York.

Legler, J. M. (1964): A narrow-mouthed toad (*Gastrophryne usta*) in El Salvador. Herpetologica 19: 286-287.

Lehtinen, R. M. (2003): Parental care and reproduction in two species of *Mantidactylus* (Anura: Mantellidae). Journal of Herpetology 37: 766-768.

Lemos-Espinal, J. A. & J. R. Dixon (2013): Amphibians and reptiles of San Luis Potosi. Eagle Mountain Publishing, Eagle Mountain.

Lescure, J. & C. Marty (2000): Atlas des amphibiens de Guyane. Collections Patrimoines Naturels. Paris 45: 1-388.

Lima, A. P., W. E. Magnusson, M. Menin, L. K. Erdtmann, D. J. Rodrigues, C. Keller & W. Hödl (2005): Guia de Sapos da Reserva Adolpho Ducke, Amazonia Central. Instituto Nacional de Pesquisas da Amazônia, Manaus.

Lisboa, B. S., F. A. C. do Nascimento & G. O. Skuk (2011): Redescription of the tadpole of *Macrogenioglottus alipioi* (Anura: Cycloramphidae), a rare and endemic species of the Brazilian Atlantic Forest. Zootaxa 3046: 67-68.

Loader, S. P., G. J. Measey, R. O. de Sa & P. K. Malonza (2009): A new brevicipitid species (Brevicipitidae: *Callulina*) from the fragmented forests of the Taita Hills, Kenya. Zootaxa 2123: 55-68.

Lobo, F. (1994): Descripción de una nueva especie de *Pseudopaludicola* (Anura: Leptodactylidae), redescripción de *P. falcipes* (Hensel, 1867) y *P. saltica* (Cope, 1887) y osteología de las tres especies. Cuadernos de herpetología 8: 177-199.

Lötters, S., W. Haas, S. Schick & W. Böhme (2002): On the systematics of the harlequin frogs (Amphibia: Bufonidae: Atelopus) from Amazonia. II: Redescription of *Atelopus pulcher* (Boulenger, 1882) from the eastern Andean versant in Peru. Salamandra 38: 165-184.

Lötters, S., K.-H. Jungfer, F. W. Henkel & W. Schmidt (2007): Pfeilgiftfrösche - Biologie, Haltung, Arten. Edition Chimaira, Frankfurt/M.

Lucas, E. M., C. A. Brasileiro, H. M. Oyamaguchi & M. Martins (2008): The reproductive ecology of *Leptodactylus fuscus* (Anura, Leptodactylidae): new data from natural temporary ponds in the Brazilian Cerrado and a review throughout its distribution. Journal of Natural History 42: 2305-2320.

Lynch, J. D. (1968): Systematic status of some Andean leptodactylid frogs with a description of a new species of *Eleutherodactylus*. Herpetologica 24: 289-300.

Lynch, J. D. (1969): Identity of two Andean *Eleutherodactylus* with the description of a new species (Amphibia: Leptodactylidae). Journal of Herpetology 33: 135-143.

Lynch, J. D. (1970): A taxonomic revision of the leptodactylid frog genus *Syrrhophus* Cope. University of Kansas Publications, Museum of Natural History 20: 1-45.

Lynch, J. D. (1975): A review of the broad-headed eleutherodactyline frogs of South America (Leptodactylidae). Occasional Papers of the Museum of Natural History, The University of Kansas 38: 1-46.

Lynch, J. D. (1984) New frogs (Leptodactylidae: *Eleutherodactylus*) from cloud forests in the Northern Cordillera Oriental, Colombia. Milwakee Public Museum. Contributions in Biology and Geology 60: 1-19.

Lynch, J. D. (1994): Two new species of the *Eleutherodactylus* conspicillatus group (Amphibia: Leptodactylidae) from the Cordillera Oriental of Colombia. Revista de la Academia Colombiana de Ciencias Exactas, Físicas y Naturales 19: 187-193.

Lynch, J. D. (1998): New species of *Eleutherodactylus* from the Cordillera Occidental of western Colombia with a synopsis of the distribution of species in western Colombia. Revista de la Academia Colombiana de Ciencias Exactas, Físicas y Naturales 22: 117-148.

Lynch, J. D. (2000): The relationships of an ensemple of Guatemalan and Mexican frogs (*Eleutherodactylus*: Leptodactylidae: Amphibia). Revista de la Academia Colombiana de Ciencias Exactas, Físicas y Naturales 24: 129-156.

Lynch, J. D. (2001): Three new rainfrogs of the *Eleutheropdactylus diastema* group from Colombia and Panama. Revista de la Academia Colombiana de Ciencias Exactas, Físicas y Naturales 25: 287-297.

Lynch, J. D. & M. C. Ardila-Robayo (1999): The *Eleutherodactylus* of the taeniatus complex in western Colombia: taxonomy and distribution. Revista de la Academia Colombiana de Ciencias Exactas, Físicas y Naturales 23: 615-624.

Lynch, J. D. & W. E. Duellman (1980): The Eleutherodactylus of the Amazonian slopes of the Ecuadorian Andes (Anura: Leptodactylidae). The University of Kansas. Museum of Natural History. Miscellaneous Publications. 69: 1-86.

Lynch, J. D. & W. E. Duellman (1997): Frogs of the Genus Eleutherodactylus in Western Ecuador. Systematics, Ecology, and Biogeography. The University of Kansas. Museum of Natural History. Special Publications. 23: 1-236.

Lynch, J. D. & M. S. Hoogmoed (1977): Two new species of Eleutherodactylus (Amphibia: Leptodactylidae) from northeastern South America. Proceedings of the Biological Society of Washington 90: 424-439.

Lynch, J. D. & L. Trueb (1980): A new species of Eleutherodactylus (Leptodactylidae) from the cloud forests of western Ecuador. Copeia 1980: 392-396.

Maffei, F. & F. K. Ubaid (2014): Amphibians of Rio Claro Farm. Lençóis Paulista, São Paulo, Brazil. Canal 6, Bauru, SP. Maiditsch, I., H. C. Liedtke, J. M. Ng’wava & W. Hoedl (2011): Advertisement and close-range encounter call of *Arthroleptis schubotzi* Nieden, 1911, with notes on phonotaxis and sexual dimorphism in the third manual digit. Herpetozoa 24: 23-31.

Malkmus, R. & J. M. Dehling (2008): Anuran amphibians of Borneo as phytotelm-breeders—a synopsis. Herpetozoa 20: 165-172.

Malkmus, R., U. Manthey, G. Vogel, P. Hoffmann & J. Kosuch (2002): Amphibians and reptiles of Mount Kinabalu (North Borneo). A.R.G. Gantner Verlag K.G, Ruggell.

Malone, J. H. (2004): Reproduction in three species of *Smilisca* from Costa Rica. Journal of Herpetology 38: 27-35.

Manamendra-Arachchi, K. & D. Gabadage (1996): *Limnonectes kirtisinghei*, a new species of ranid frog from Sri Lanka. Journal of South Asian natural History 2: 31-42.

Manamendra-Arachchi, K. & R. Pethiyagoda (2005): The Sri Lankan shrub-frogs of the genus *Philautus* Gistel, 1848 (Ranidae: Rhacophorinae), with description of 27 new species. Raffles Bulletin of Zoology 12: 163-303.

Manthey, U. & W. Grossmann (1997): Amphibien & Reptilien Südostasiens. Natur und Tier Verlag, Münster.

Marin da Fonte, L. F. (2010): Variacao morfológica e na estrutura do canto em *Scinax granulatus* (Peters, 1871) (Anura, Hylidae). Universidade Federal do Rio Grande do Sul, Porto Alegre.

Martins, I. A. (2001): Parental care behaviour in *Leptodactylus podicipinus* (Cope, 1862) (Anura, Leptodactylidae). Herpetological Journal 11: 29-32.

Mathe, R. & N. Sen (2010): Pictorial guide to amphibians of North East India. Zoological Survey of India, Kolkata.

Measey, G. J., P. K. Malonza & V. Muchai (2009): Amphibians of the Taita Hills. SANBI Biodiversity Series 12. South African National Biodiversity Institute, Pretoria.

Mendelson III, J. R. (1997): A new species of toad (Anura: Bufonidae) from Oaxaca, Mexico with comments on the status of *Bufo cavifrons* and *Bufo cristatus*. Herpetologica 53: 268-286.

Menin, M., D. J. Rodrigues & A. P. Lima (2007): Clutches, tadpoles and advertisement calls of *Synapturanus mirandaribeiroi* and *S. cf. salseri* in Central Amazonia, Brazil. The Herpetological Journal 17: 86-91.

Mercurio, V. (2011): Amphibians of Malawi. An analysis of the richness and community diversity in a changing landscape. Edition Chimaira, Frankfurt/M.

Mercurio, V., W. Böhme & B. Streit (2009): Reproductive diversity of Malawian anurans. Herpetology Notes 22: 175-183.

Mertens, R. (1965): Die Amphibian von Fernando Poo. Bonner Zoologische Beiträge 16: 14-29.

Minter, L. R., M. Burger, J. A. Harrison, H. H. Braack, B. P. J. & D. Knoepfer (2004): Atlas and red data book of the frogs of South Africa, Lesotho & Swaziland. SI/MAB Biodiversity Program Series #9, Smithsonian Institution, Washington D.C.

Moura, G. J. B., E. V. E. Andrade & E. M. X. Freire (2010): Amphibia, Anura, Microhylidae, *Stereocyclops incrassatus* Cope, 1870: Distribution extension. Check List 6: 71-72.

Muniz, K. P. R., A. A. Giaretta, W. R. Silva & K. G. Facure (2008): Auto-ecologia de *Hypsiboas albopunctatus* (Anura, Hylidae) em área de Cerrado no sudeste do Brasil. Iheringia, Série Zoologia 98: 254-259.

Murrieta-Galindo, R., A. González-Romero, F. López-Barrera & G. Parra-Olea (2013): Coffee agrosystems: an important refuge for amphibians in central Veracruz, Mexico. Agroforestry Systems 87: 1-13.

Myers, C. W. (1969): The ecological geography of cloud forest in Panama. American Museum novitates 2396: 1-52.

Myers, C. W. & M. A. Donnelly (2001): Herpetofauna of the Yutaje-Corocoro massif, Venezuela: second report from the Robert G. Goelet American Museum-Terramar expedition to the northwestern Tepuis. Bulletin of the American Museum of Natural History 261: 1-85.

Nair, A., S. V. Gopalan, S. George, K. S. Kumar, A. G. F. Teacher & J. Merilä (2012): Endemic Indirana frogs of the Western Ghats biodiversity hotspot. Annales Zoologici Fennici 49: 257-286.

Narzary, J. & S. Bordoloi (2013): Study of normal development and external morphology of tadpoles of *Microhyla ornata* and *Uperodon globulosus* of the family Microhylidae (Amphibia: Anura) from North East India. Internatational Journal of Advanced Biological Research 3: 61-73.

Nascimento, L. B., B. V. S. Pimenta, C. A. G. Cruz & U. Caramaschi (2006): Taxonomic status of *Gomphobates marmoratus* Reinhardt and Lütken, 1862 “1861” and *Eupemphix fuscomaculatus* Steindachner, 1864 (Amphibia, Anura, Leptodactylidae). South American Journal of Herpetology 1: 166-174.

Norman, D. R. (1994): Anfibios y Reptiles del Chaco Paraguayo, Tomo 1. (Amphibians and reptiles of the Paraguayan Chaco, Volume 1.). Private printing, San José, Costa Rica.

Ohler, A., S. R. Swan & J. C. Daltry (2002): A recent survey of the amphibian fauna of the Cardomom Mountains, Southwest Cambodia with descriptions of three new species. Raffles Bulletin of Zoology 50: 465-482.

Orlow, N. L., S. A. Ryabov, N. B. Ananjeva & A. A. Evsyunin (2010): Asian treefrogs of the genus *Theloderma* Tschudi, 1838 (Amphibia: Anura: Rhacophoridae: Rhacophorinae). Russian Academy of Sciences, Zoological Institute, St. Petersburg.

Ouboter, P. E. & R. Jairam (2012): Amphibians of Suriname. Brill, Leiden.

Peracca, D. M. G. (1904): Rettili ed Amfibii. Facsimiles. Bollettino dei Musei di Zoologia ed Anatomia comparata della R. Università di Torino 19: 429-469.

Perret, J.-L. (1966): Les amphibiens du Cameroun. Zoologische Jahrbücher (Systematik) 8: 289-464.

Perret, J.-L. (1977): Les Hylarana (Amphibiens, Ranidés) du Cameroun. Revue suisse de Zoologie 84: 841-868.

Perret, J.-L. (1983): Nouvelles données sur Hylarana occidentalis Perret (Amphibia, Ranidae). Bulletin de la Société Neuchâteloise des Sciences Naturelles 106: 109-113.

Perret, J.-L. (1988): Les espèces de Phrynobatrachus (Anura, Ranidae) à éperon palpébral. Archives des Sciences Genève 41: 275-294.

Pickersgill, M. (2007): A redefinition of Afrixalus fulvovittatus (Cope, 1860) and Afrixalus vittiger (Peters, 1876) (Amphibia, Anura Hyperoliidae). African Journal of Herpetology 56: 23-37.

Pillai, R. S. & R. Pattabiraman (1990): Amphibians from Sabarigiri forest, Western Ghats, Kerala, including a new species of *Micrixalus*. Records of Zoological Survey India 86: 383-390.

Pimenta, B. V. S., C. A. G. da Cruz & M. Dixo (2002): *Chiasmocleis carvalhoi*. Herpetological Review 33: 219.

Pineda, E. & C. A. Rodríguez-Mendoza (2010): Distribución y abundancia de Craugastor vulcani: una especie de rana en riesgo de Los Tuxtlas, Veracruz, México. Revista mexicana de biodiversidad 81: 133-141.

Poynton, J. C. (1964): Amphibia of southern Africa; a faunal study. Annals of the Natal Museum 17: 1-334.

Preininger, D., M. Boeckle, A. Freudmann, I. Starnberger, M. Sztatecsny & W. Hödl (2013): Multimodal signaling in the small torrent frog (*Micrixalus saxicola*) in a complex acoustic environment. Behavioral ecology and sociobiology 67: 1449-1456.

Proy, C. (1992): Zur Biologie von *Agalychnis saltator* Taylor, 1955. Herpetozoa 5: 99-107.

Pupin, N. C., J. L. Gasparini, R. P. Bastos, C. F. B. Haddad & C. de Almeida Prado (2010): Reproductive biology of an endemic *Physalaemus* of the Brazilian Atlantic forest, and the trade-off between clutch and egg size in terrestrial breeders of the *P. signifer* group. The Herpetological Journal 20: 147-156.

Rabb, G. B. & M. S. Rabb (1963): On the behavior and breeding biology of the African pipid frog: *Hymenochirus boettgeri*. Zeitschrift für Tierpsychologie 20: 215-241.

Randrianiaina, R.-D., L. Raharivololoniaina, C. Preuss, A. Strauss, F. Glaw, M. Teschke, J. Glos, N. Raminosoa & M. Vences (2009): Descriptions of the tadpoles of seven species of Malagasy treefrogs, genus *Boophis*. Zootaxa 2021:23-41.

Randrianiaina, R. D., K. C. Wollenberg, T. Rasolonjatovo Hiobiarilanto, A. Strauß, J. Glos & M. Vences (2011): Nidicolous tadpoles rather than direct development in Malagasy frogs of the genus *Gephyromantis*. Journal of Natural History 45: 2871-2900.

Rivero, J. A. (1971): Tres nuevos records y una nueva especie de anfibios de Venezuela. Caribbean Journal of Science 11: 1-9.

Roberto, I. J., L. Brito & P. Cascon (2011): Temporal and spatial patterns of rerpoductive activity in *Rhinella hoogmoedi* (Anura: Bufonidae) from a tropical rainforest in Northeastern Brazil, with the description of it’s advertisement call. South American Journal of Herpetology 6: 87-97.

Rödel, M.-O. (2000): Herpetofauna of West Africa. Vol I Amphibians of the West African savanna. Edition Chimaira, Frankfurt.

Rödel, M.-O. & W. R. Branch (2002): Herpetological survey of the Haute Dodo and Cavally forests, western Ivory Coast, Part I: amphibians. Salamandra 38: 245-268.

Rödel, M.-O. & R. Ernst (2001): Description of the tadpole of *Kassina lamottei* Schiøtz, 1967. Journal of Herpetology 35: 678-681.

Rödel, M.-O. & R. Ernst (2002a): A new *Phrynobatrachus* from the Upper Guinean rain forest, West Africa, including a description of a new reproductive mode for the genus. Journal of Herpetology 36: 561-571.

Rödel, M.-O. & R. Ernst (2002b): A new reproductive mode for the genus *Phrynobatrachus*: *Phrynobatrachus alticola* has nonfeeding, nonhatching tadpoles. Journal of Herpetology 36: 121-125.

Rödel, M.-O., M. Gil, A. Cudjoe Agyei, A. D. Leaché, R. E. Diaz, M. K. Fuujita & R. Ernst (2005): The amphibians of the forested parts of south-western Ghana. Salamandra 41: 107-127.

Rödel, M.-O., J. Kosuch, M. Veith & R. Ernst (2003): First record of the genus *Acanthixalus* Laurent, 1944 from the upper Guinean rain forest, West Africa, with the description of a new species. Journal of Herpetology 37: 43-52.

Rödel, M.-O., D. Krätz & R. Ernst (2002a): The tadpole of *Ptychadena aequiplicata* (Werner, 1898) with the description of a new reproductive mode for the genus (Amphibia, Anura, Ranidae). Alytes 20: 1-12.

Rödel, M.-O., F. Range, J.-T. Seppänen & R. Noë. (2002b): Caviar in the rain forest: monkeys as frog-spawn predators in Taï National Park, Ivory Coast. Journal of Tropical Ecology 18: 289-294.

Rödel, M.-O., V. H. W. Rudolf, S. Frohschammer, K. E. Linsenmair & R. M. Lehtinen (2004a): Life history of a West-African tree-hole breeding frog, *Phrynobatrachus guineensis* Guibé and Lamotte, 1961 (Amphibia: Anura: Petropedetidae). Miscellaneous Publications Museum of Zoology University of Michigan 193: 31-44.

Rödel, M.-O., A. Schmitz, O. S. G. Pauwels & W. Böhme (2004b): Revision of the genus *Werneria* Poche, 1903, including the description of two new species from Cameroon and Gabon (Amphibia: Anura: Bufonidae). Zootaxa 720: 1-28.

Rödel, M.-O. & M. Spieler (2000): Trilingual keys to the savannah-anurans of the Comoé National Park, Ivory Coast. Stuttgarter Beitrage zur Naturkunde Serie A 620: 1-31.

Rodrigues, D. J., M. Menin & A. P. Lima. 2007. Redescription of the tadpole of *Leptodactylus rhodomystax* (Anura: Leptodactylidae) with natural history notes. Zootaxa 1509: 61-67.

Rodrigues, D. J., M. Uetanabaro & F. S. Lopes (2005): Reproductive patterns of *Trachycephalus venulosus* (Laurenti, 1768) and *Scinax fuscovarius* (Lutz, 1925) from the Cerrado, Central Brazil. Journal of Natural History 39: 3217-3226.

Rodriguez, L. O. & W. E. Duellman (1994): Guide to the frogs of the Iquitos region, Amazonian Peru. Natural History Museum, University of Kansas, Lawrence.

Roelke, C. E., E. Greenbaum, C. Kusamba, M. M. Aristote & E. N. Smith (2011): Systematics and conservation status of two distinct Albertine Rift treefrogs, *Leptopelis karissimbensis* and *Leptopelis kivuensis* (Anura: Arthroleptidae). Journal of Herpetology 45: 343-351.

Rojas-Rivera, A., S. Cortés-Bedoya & P. D. A. Gutiérrez-Cárdenas (2011): Pristimantis achantinus (cachabi robber frog). Parental care and clutch size. Herpetological Review 42: 588-589.

Roux, J. (1904): Reptilien und Amphibien aus Celebes. Verhandlungen der Naturforschenden Gesellschaft in Basel 15: 425-433.

Ruas, D. S., C. V. M. Mendes, B. B. Szpeiter & M. Sole (2012): The tadpole of Rhinella crucifer (Wied-Neuwied, 1821) (Amphibia: Anura: Bufonidae) from southern Bahia, Brazil. Zootaxa 3299: 66-68.

Rueda Almonacid, J. V., J. V. Rodriguez Mahecha, E. La Marca, S. Lötters, T. Kahn & A. Angulo (eds.) (2005): Ranas Arlequines. Conservación Internacional.

Sabater-Pi, J. (1984): Contribution to the biology of the giant frog (*Conraua goliath*, Boulenger). Amphibia-Reptilia 6: 143-153.

Sánchez, D. A. (2010): Larval development and synapomorphies for species groups of *Hyloscirtus* Peters, 1882 (Anura: Hylidae: Cophomantini). Copeia 2010: 351-363.

Santana, D. J., A. P. Motta, R. M. Pirani, E. T. da Silva & R. N. Feio (2012): Advertisement call and tadpole of *Chiasmocleis mantiqueira* Cruz, Feio and Cassini, 2007 (Anura, Microhylidae). Journal of Herpetology 46: 14-18.

Sarkar, A. K. & D. P. Sanyal (1985): Amphibia. Records of Zoological Survey India 82: 285-295.

Savage, J. M. (1984): A new species of montanae rain frog, genus *Eleutherodactylus* (Leptodactylus) from Guerrero, Mexico. Amphibia-Reptilia 5: 253-260.

Savage, J. M. (2002): The amphibians and reptiles of Costa Rica. University of Chicago Press, Chicago.

Schiøtz, A. (1999): Treefrogs of Africa. Edition Chimaira, Frankfurt/M.

Schlüter, A. & A. W. Salas (1991): Reproduction, tadpoles, and ecological aspects of three syntopic microhylid species from Peru (Amphibia: Microhylidae). Stuttgarter Beiträge zur Naturkunde 458: 1-17.

Schmidt, H., A. Strauss, F. Glaw, M. Teschke & M. Vence. (2009): Description of tadpoles of five frog species in the subgenus *Brygoomantis* from Madagascar (Mantellidae: Mantidactylus). Zootaxa 1988: 48-60. Shimizu, S. & H. Ota (2003): Normal development of *Microhyla ornata*: the first description of the complete embryonic and larval stages for the microhylid frogs (Amphibia: Anura). Current Herpetology 22: 73-90.

Smith, H. M. (1939): Mexican herpetological novelties. Proceedings of the Biological Society of Washington 52: 187-196.

Sokol, O. M. (1962): The tadpole of *Hymenochirus boettgeri*. Copeia 1962: 272-284.

Spawls, S., K. Howell & R. C. Drewes (2006): Pocket guide to the reptiles and amphibians of East Africa. A. & C. Black., London.

Staniszewski, M. (2001): Mantellas. Edition Chimaira, Frankfurt/M.

Stewart, M. M. (1967): The amphibians of Malawi. State University Press, New York.

Tapley, B., C. Babburjung Purushotham & S. Girgin (2011): *Indirana semipalmata* (brown leaping frog). Reproduction. Herpetological Review 42: 87-88.

Targino, M. & J. P. Pombal (2011): Redescription and variation of *Hyophryne histrio* Carvalho, 1954, an enigmatic microhylid frog from the Atlantic Rainforest of Brazil. Amphibia-Reptilia 32: 465-475.

Taylor, E. H. (1938): New species of Mexican tailless amphibia. The University of Kansas. Science Bulletin 25: 285-405.

Taylor, E. H. (1950): A new bromeliad frog from the Mexican state of Veracruz. Copeia 1950: 274-276.

Taylor, E. H. (1962): The amphibian fauna of Thailand. University of Kansas Science Bulletin 43: 265-599.

Teixeira, R. L., D. Vrcibradic & G. I. Almeida (2006): Food habits of *Stereocyclops incrassatus* (Anura, Micohylidae) from Povoação, Espírito Santo State, southeastern Brazil. Boletim do Museu Mello Leitão 19: 53-58.

Tessa, G., F. Mattioli, V. Mercurio & F. Andreone (2009): Egg numbers and fecundity traits in nine species of *Mantella* poison frogs from arid grasslands and rainforests of Madagascar (Anura: Mantellidae). Madagascar Conservation & Development 4: 113-119.

Toledo, L. F., L. M. Castanho & C. F. B. Haddad (2005): Recognition and distribution of *Leptodactylus mystaceus* (Anura; leptodactylidae) in the state of São Paulo, southeastern Brazil. Biota Neotropica 5: 57-62.

Trueb, L. & D. Massemin (2001): The osteology and relationships of *Pipa aspera* (Amphibia: Anura: Pipidae), with notes on its natural history in French Guiana. Amphibia-Reptilia 22: 33-54.

Truong, N. Q. & N. V. Khoi (2008): First record of *Theloderma stellatum* Taylor, 1962 from Phu Quoc Island, Kien Giang Province, southern Vietnam. Herpetology Notes 1: 61-62.

Tyler, M. J. & F. Knight (2009): Field guide to the frogs of Australia. CSIRO Publishing, Collingwood, Victoria.

Ukuwela, K. D. B. (2009): Description of the tadpole of *Hylarana aurantiaca* (Anura: Ranidae) from Sri Lanka. Zootaxa 2016: 67-68.

Ukuwela, K. D. B. & I. N. Bandara (2009): The first description of the tadpole of *Lankanectes corrugatus* (Peters, 1864) (Anura: Nyctibatrachidae) from Sri Lanka. Russian Journal of Herpetology 16: 213-216.

Urbina-Cardona, J. N. & V. H. Reynoso (2009): Uso del microhábitat por hembras grávidas de la rana de hojarasca *Craugastor loki* en la selva alta perennifolia de Los Tuxtlas, Veracruz, México. Revista mexicana de biodiversidad 80: 571-573.

Veeranagoudar, D. K., R. S. Radder, B. A. Shanbhag & S. K. Saidapur (2009): Jumping behavior of semiterrestrial tadpoles of *Indirana beddomii* (Günth.): relative importance of tail and body size. Journal of Herpetology 43: 680-684.

Veeranagoudar, D. K., B. A. Shanbhag & S. K. Saidapur (2004): Foraging behaviour in tadpoles of the bronze frog *Rana temporalis*: experimental evidence for the ideal free distribution. Journal of Biosciences 29: 201-207.

Vejarano, S., M. Thomas & M. Vences (2006): Comparative tadpole morphology in three species of frogs of the genus *Spinomantis* (Amphibia: Mantellidae). Contributions to Zoology 75: 99-108.

Venâncio, N. M. & P. R. Melo-Sampaio (2010): Reproductive behavior of the giant leaf frog *Phyllomedusa bicolor* (Anura: Hylidae) in the western Amazon. Phyllomedusa 9: 63-67.

Vences, M., F. Glaw & C. Zapp (1999): Bemerkungen zu Eizahlen und Eigrößen bei madagassischen Fröschen der Gattungen *Tomopterna*, *Aglyptodactylus*, *Boophis* und *Mantidactylus* (Amphibia: Ranidae). Salamandra 35: 77-82.

Vences, M., C. J. Raxworthy, R. A. Nussbaum & F. Glaw (2003): New microhylid frog (Plethodontohyla) from Madagascar, with semiarboreal habits and possible parental care. Journal of Herpetology 37: 629-636.

Verdade, V. K. & M. T. Rodrigues (2007): Taxonomic review of *Allobates* (Anura, Aromobatidae) from the Atlantic Forest, Brazil. Journal of Herpetology 41: 566-580.

Vitt, L. J. & J. P. Caldwell (2013): Herpetology: an introductory biology of amphibians and reptiles. 4 edition. Academic Press, London.

Vonesh, J. R. & S. Ross. (2000): Dipteran predation on the arboreal eggs of four Hyperolius frog species in western Uganda. Copeia 2000: 560-566.

Wang, Y.-Y., T.-D. Zhang, J. Zhao, Y.-H. Sung, J.-H. Yang, H. Pang & Z. Zhang (2012): Description of a new species of the genus Xenophrys Günther, 1864 (Amphibia: Anura: Megophryidae) from Mount Jinggang, China, based on molecular and morphological data. Zootaxa 3546: 53-67.

Werner, F. (1899): Über Reptilien und Batrachier aus Columbien und Trinidad. Verhandlungen der kaiserlichköniglichen zoologisch-botanischen Gesellschaft in Wien 49: 470-484.

Wickramasinghe, D. D., K. L. Oseen, S. W. Kotagama & R. J. Wassersug (2004): The terrestrial breeding biology of the ranid rock frog *Nannophrys ceylonensis*. Behaviour 141: 899-913.

Wickramasinghe, L. J. M., D. R. Vidanapathirana, M. D. G. Rajeev, S. C. Ariyarathne, A. W. A. Chanaka, L. L. D. Priyantha, I. N. Bandara & N. Wickramasinghe (2013): Eight new species of *Pseudophilautus* (Amphibia: Anura: Rhacophoridae) from Sripada World Heritage Site (Peak Wilderness), a local amphibian hotspot in Sri Lanka. Journal of Threatened Taxa 5: 3789-3920.

Wildenhues, M. J., A. Gawor, T. Q. Nguyen, T. T. Nguyen, A. Schmitz & T. Ziegler (2010): First description of larval and juvenile stages of *Rhacophorus maximus* Gunther, 1859 "1858" (Anura: Rhacophoridae) from Vietnam. Revue suisse de Zoologie 117: 679-696.

Wilkinson, J. A., T. Thin, K. S. Lwin & A. K. Shein (2005): A new species of *Rhacophorus* (Anura: Rhacophoridae) from Myanmar (Burma). Proceedings-California Academy of Sciences 56: 42-52.

Wogan, G. O. U. (2012): A new species of *Leptobrachium* from Myanmar (Anura: Megophryidae). Zootaxa 3415: 23-36.

Wolf, S. (2013): A case of male egg guarding behaviour in a stream-dwelling frog, *Mantidactylus* (Ochthomantis) sp (Anura: Mantellidae), from northeastern Madagascar. Salamandra 49: 45-47.

Zina, J., G. R. Silva, D. Loebmann & V. G. D. Orrico (2014): The recognition of *Dendropsophus minusculus* (Rivero, 1971) (Hylidae, Dendropsophini) as a highly polymorphic, multi-domain distributed species. Brazilian Journal of Biology 74: 146-153.

**Webpages:**

African amphibians Lifedesk http://africanamphibians.lifedesks.org/

Amphibia Web http://amphibiaweb.org/

Amphibians of Panama http://biogeodb.stri.si.edu/amphibians/en/

Amphibiaweb http://amphibiaweb.org/

Animal Diversity Web http://animaldiversity.ummz.umich.edu

Dendroworld http://www.dendroworld.co.uk

Frog Atlas http://www.frogatlas.com.au/

Frog Forum http://www.frogforum.net/content/

Frog.org http://talkto.thefrog.org/

Frogs of Borneo http://frogsofborneo.org/

Hepetofauna Costa Ricas http://www.herpetologia.de

IUCN http://www.iucnredlist.org/

Online field guide http://online-field-guide.com/

Reptiles http://www.reptilesmagazine.com/

Tropical Herping http://www.tropicalherping.com/default.html

Wikipedia <https://en.wikipedia.org>
