# Supplementary material for: What makes a successful species? Traits facilitating survival in altered tropical forests
Source: BMC Ecol. 2017 Jun 28;17:25. doi: 10.1186/s12898-017-0135-y (PMC5490239; doi:10.1186/s12898-017-0135-y)
Supplement: Supplementary file 3 — Additional file 3. Clutch size classes. Additional methods describing the objective grouping of clutch sizes. [file 12898_2017_135_MOESM3_ESM.docx]

**Additional file 3. Clutch size classes**

Clutch size was only available for a subset (345 out of 619) of species. An objective grouping by applying K-means clustering (*kmeans*, R package ‘stats’; 1000 iterations) on the available data revealed the following clutch size classes (mean, range, sample size = species with this clutch size): A: 38.6, 4-98, n = 121; B: 160.5, 100-265, n = 68, C: 392.0, 290-549, n =37; D: 712.0, 563-905, n = 24; E: 1201.6, 979-1652, n =37; F: 2408.7, 1900-3320, n = 32, G: 4724.5, 3607-6701, n = 13; H: 10420.0, 8357-12940, n = 5; I: 21400.0, 17000-25000, n = 5, J: 38050.0, 36100-40000, n = 2. Ten classes were chosen to achieve a fine scale fractionation but at the same time at least a number of five records per class; except for the biggest size class. Species without information on clutch size were subsequently assigned to a single class based female (or male if female size was not available) body size, as the maximal number of eggs per clutch was significantly correlated with body size (females: rho = 0.58, p < 0.0001, n = 334; males: rho = 0.58, p < 0.0001, n = 341). Crucial was the minimal distance of the body size of a species lacking information to the mean body size of a particular clutch size class. 266 species were assigned based on female, 34 based on male body size. Calculation of clutch size classes and the assignment to a category was conducted independently of the reproductive mode, i.e. direct and indirect developing species together. This was permitted as data distribution of clutch size as well as the data distribution of clutch size related to female body size did not differ between the modes, i.e. data of direct developing species were within the range of clutch sizes of indirect developing species with similar body size (Kolmogorov-Smirnov test: clutch size: D = 0.08, p = 0.23; clutch size / female body size: D = 0.07, p = 0.46; N: direct developing species = 47, indirect developing species = 297; see Figure below).


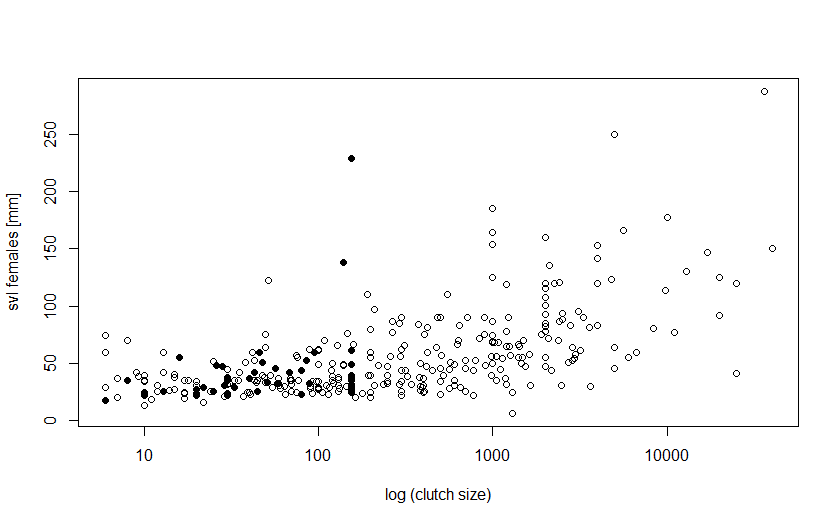


Given are the maximum clutch size (log transformed) and the maximum female body size (snout vent length, svl) of 344 species. Direct developing species (n = 47) are marked with black, indirect developing species (n = 297) with white dots
