# Supplementary material for: What makes a successful species? Traits facilitating survival in altered tropical forests
Source: BMC Ecol. 2017 Jun 28;17:25. doi: 10.1186/s12898-017-0135-y (PMC5490239; doi:10.1186/s12898-017-0135-y)
Supplement: Supplementary file 5 — Additional file 5. Affiliation to different anuran families. Number and relative frequency of species belonging to a particular family per forest dependency index. [file 12898_2017_135_MOESM5_ESM.docx]

**Additional file 5. Affiliation to different anuran families**

Given are number and relative frequency (in color code) of species belonging to a particular family per forest dependency index (higher frequency with darker color, see legend in table); forest dependency index: D = dependent (n = 33), SD = slightly dependent (n = 108), NR = non-responding (n = 83), I = forest independent (n = 19).

| Family | Forest dependency Index | | | |  |  |
| --- | --- | --- | --- | --- | --- | --- |
|  | D | SD | NR | I |  |  |
| Aromobatidae | 0 | 2 | 0 | 0 |  |  |
| Arthroleptidae | 2 | 9 | 3 | 0 |  |  |
| Brevicipitidae | 1 | 0 | 0 | 1 |  |  |
| Bufonidae | 2 | 2 | 10 | 1 |  |  |
| Centrolenidae | 1 | 1 | 0 | 0 |  |  |
| Ceratophryidae | 0 | 1 | 0 | 0 |  |  |
| Craugastoridae | 1 | 7 | 6 | 0 |  |  |
| Dendrobatidae | 0 | 0 | 1 | 0 |  |  |
| Dicroglossidae | 1 | 3 | 6 | 2 |  |  |
| Eleutherodactylidae | 0 | 1 | 2 | 0 |  |  |
| Hemisotidae | 0 | 1 | 1 | 0 |  |  |
| Hylidae | 4 | 13 | 15 | 4 |  |  |
| Hyperoliidae | 4 | 4 | 1 | 1 |  |  |
| Leiuperidae | 0 | 0 | 1 | 0 |  |  |
| Leptodactylidae | 0 | 4 | 8 | 0 |  |  |
| Mantellidae | 0 | 31 | 4 | 4 |  |  |
| Megophryidae | 0 | 1 | 0 | 0 |  |  |
| Microhylidae | 1 | 14 | 6 | 1 |  |  |
| Petropedetidae | 0 | 2 | 0 | 0 |  |  |
| Phrynobatrachidae | 0 | 1 | 2 | 1 |  |  |
| Pipidae | 2 | 0 | 0 | 0 |  |  |
| Ptychadenidae | 1 | 1 | 2 | 0 |  |  |
| Pyxicephalidae | 2 | 2 | 0 | 1 |  | 0 |
| Ranidae | 0 | 5 | 7 | 0 |  | < 10% |
| Ranixalidae | 0 | 1 | 1 | 0 |  | < 30% |
| Rhacophoridae | 11 | 2 | 7 | 3 |  | > 30% (33%) |
